# A novel network-based method identifies a cuproplasia-related pan-cancer gene signature to predict patient outcome

**DOI:** 10.1007/s00439-024-02673-2

**Published:** 2024-04-20

**Authors:** Vu Viet Hoang Pham, Toni Rose Jue, Jessica Lilian Bell, Fabio Luciani, Filip Michniewicz, Giuseppe Cirillo, Linda Vahdat, Chelsea Mayoh, Orazio Vittorio

**Affiliations:** 1 Children’s Cancer Institute, Lowy Cancer Research Centre, UNSW, Kensington, NSW Australia; 2 School of Biomedical Sciences, UNSW Sydney, Kensington, NSW Australia; 3 Department of Pharmacy, Health and Nutritional Sciences, University of Calabria, Rende, Italy; 4 Dartmouth–Hitchcock Medical Center: Lebanon, New Hampshire, US; 5 School of Clinical Medicine, UNSW Medicine & Health, UNSW Sydney, Kensington, NSW Australia

**Keywords:** Cuproplasia, Copper, Cancer, Gene regulatory network, Survival analysis

## Abstract

Copper is a vital micronutrient involved in many biological processes and is an essential component of tumour cell growth and migration. Copper influences tumour growth through a process called cuproplasia, defined as abnormal copper-dependent cell-growth and proliferation. Copper-chelation therapy targeting this process has demonstrated efficacy in several clinical trials against cancer. While the molecular pathways associated with cuproplasia are partially known, genetic heterogeneity across different cancer types has limited the understanding of how cuproplasia impacts patient survival. Utilising RNA-sequencing data from The Cancer Genome Atlas (TCGA) and the Genotype-Tissue Expression (GTEx) datasets, we generated gene regulatory networks to identify the critical cuproplasia-related genes across 23 different cancer types. From this, we identified a novel 8-gene cuproplasia-related gene signature associated with pan-cancer survival, and a 6-gene prognostic risk score model in low grade glioma. These findings highlight the use of gene regulatory networks to identify cuproplasia-related gene signatures that could be used to generate risk score models. This can potentially identify patients who could benefit from copper-chelation therapy and identifies novel targeted therapeutic strategies.

**Supplementary Information:**

The online version contains supplementary material available at 10.1007/s00439-024-02673-2.

## Introduction

Cuproplasia is defined as copper-dependent cell-growth and proliferation in disease (Ge et al. 2022), which is distinct from cuproptosis, a process in which copper mediates cell death via cytotoxicity caused by increased mitochondrial-dependent energy metabolism and accumulation of reactive oxygen species (Tang et al. 2022). This definition of cuproplasia was introduced at the Copper Cancer Consortium at Cold Spring Harbour Laboratory in 2020 (Ge et al. 2022). Cuproplasia can be pharmacologically targeted; meaning that copper signalling can be activated with metal ionophores or repressed by copper-selective chelators. It can also be modulated by genetic manipulation of proteins related to copper homeostasis, which is regulated by the copper uptake, transport, and excretion within the cell and individual intracellular compartments.

Copper is a metal involved in many biological processes and is essential for cell growth. It is a redox cofactor for several copper-dependent enzymes and proteins, such as the mitochondrial Cu transporter *SLC25A3* and cytochrome *c* oxidase *COX* (Tsang et al. 2021). It also plays an important role in angiogenesis, by influencing vascular endothelial growth factor (*VEGF*) protein and its mRNA expression (McAuslan and Reilly 1980; Sen et al. 2002). These findings highlight the indispensable role of copper as a factor for enzymes in mediating a wide range of important cellular functions. However, the dysregulation of copper stimulates receptor tyrosine kinase (RTK) signalling pathways, inhibits key enzymes in cellular metabolism and respiration (Michniewicz et al. 2021), and leads to oxidative stress and cytotoxicity (Que et al. 2008; Solomon et al. 1996). For example, cellular toxicity involved with disrupted copper homeostasis is often associated with accumulation of reactive oxygen species, anti-angiogenesis, and proteasome inhibition (Jiang et al. 2022).

Furthermore, there is evidence showing that various proteins maintain appropriate intracellular copper bioavailability in cells and ensures the metalation of copper-dependent enzymes. Key regulators of mammalian copper homeostasis include the blood carrier protein ceruloplasmin (*CP*), *SLC31A1* (also known as *CTR1*, a principal copper uptake transporter), as well as exporting proteins *ATP7A* and *ATP7B*, which possess both copper export and metallochaperone functions (Ge et al. 2022). Additionally, somatic mutations in several regulating proteins cause copper accumulation or deficiency pathologies, such as mutations in *ATP7A* disrupt the homeostatic copper balance, leading to copper deficiency in Menkes disease (de Bie et al. 2007).

With the increasing importance of understanding the role of copper and its related genes in cancer development, several studies in cancer such as in neuroblastoma (Rouaen et al. 2022), pancreatic cancer (Guan et al. 2022), glioma (Chen et al. 2022; Michniewicz et al. 2020, 2022), bladder cancer (Song et al. 2022), and breast cancer (Li et al. 2022) have investigated the pro-tumorigenic role of copper. However, cuproplasia-associated gene signatures in a pan-cancer analysis are still unknown and no signatures have been detected to show the association of cuproplasia with cancer patients’ survival. Therefore, a comprehensive pan-cancer analysis is required to identify the role of cuproplasia-related genes and their associations with survival and biological processes.

We performed a comprehensive analysis on cuproplasia-related genes in cancer to better understand their effect, potential roles, and their associations with patient survival. We utilised a network control method (Liu et al. 2011) using RNA-sequencing data from The Cancer Genome Atlas (TCGA) and Genotype-Tissue Expression (GTEx) datasets across 23 cancer types. The identified cuproplasia-related genes were correlated with patient survival and show the ability of generating a prognostic risk score model. To our knowledge, this is the first study which conducted a comprehensive pan-cancer analysis of the genes involved in cuproplasia. These results highlight the importance of identifying cuproplasia-related genes in tumour development. In addition, our findings provide novel insights on the effects of copper homeostasis on the molecular regulatory mechanisms of cancer initialisation and progression.

## Materials and methods

### Datasets and data processing

We obtained publicly available data from The Cancer Genome Atlas (TCGA) (Weinstein et al. 2013) and the Genotype-Tissue Expression (GTEx) (Lonsdale et al. 2013). From TCGA we selected 23 cancer types, based on the availability of matched RNA-sequencing data from normal tissues in GTEx (Supplementary Table 1 in Additional file 1), and patient survival data. This selection resulted in data from primary tumours and the corresponding GTEx normal tissues for 23 cancer types, for a total of 6,732 patients (6,728 patients had clinical data, including age and gender) and 4,597 normal tissues. Among the 6,728 patients, 6,678 patients had survival data and 50 patients did not include survival data. The 50 patients without survival data were removed in our survival analyses (i.e., there is no significant difference between the means of age between patients with and without survival data, *p*-values 0.5273, 0.5152, and 0.6829 for the tests of all patients, male patients, and female patients respectively using t-test). We further obtained publicly available gene expression and clinical data from a low grade glioma (LGG) dataset from the Chinese Glioma Genome Atlas (CGGA) database (http://www.cgga.org.cn/) (Liu et al. 2018b; Wang et al. 2015; Zhao et al. 2021). Only 3 patients that did not have corresponding survival data were removed from survival analyses and LGG risk score model building, resulting in a training set of 345 patients and a test cohort of 420 patients to build the LGG risk score model.

### Differentially expressed genes

Differential gene expression analysis was conducted in R (v4.2.2) using limma (Ritchie et al. 2015) to compare gene expression data (expected counts from RSEM output) of TCGA primary tumours against the matched GTEx normal tissues for the 23 cancer types. The gene expression data was downloaded from UCSC Xena browser (https://xenabrowser.net/datapages/). Genes were considered differentially expressed if they had an adjusted *p*-value ≤ 0.05 and log_2_(|fold change|) ≥ 1. Benjamini-Hochberg was used for multiple test correction.

### Construction of gene regulatory networks

Gene regulatory networks were constructed in R (v4.2.2) using Pearson correlation and STRING protein interaction database (Szklarczyk et al. 2019) for each of the 23 cancer types. In this study, Pearson correlation was utilised to identify if there was a linear relationship between two quantitative variables (i.e., the expression of two genes). However, our proposed method was flexibly designed so that end users could use Pearson or other correlation methods such as Spearman. Due to the bias of the STRING network and the fact that STRING interactions may not occur in every cancer type, we built the gene regulatory network for a cancer type using the gene expression of patients in that cancer type, then we only used the STRING database as an additional resource to refine the network. The relative gene expression values of the significantly differentially expressed genes identified by comparing primary samples with the matched normal tissues for each cancer type were filtered to only include genes in the STRING database. These genes were used for network construction. We calculated the pairwise correlation coefficients (PCC) for all the nodes and retained only edges whose adjusted *p*-value ≤ 0.05. In the network, nodes are genes and edges show the interactions between the genes. We further refined the gene network using the STRING protein interaction database, by removing interactions not in the STRING database. This resulted in the final gene regulatory network for each cancer type.

### Identification of critical nodes

We adopted established tools from network control (Liu et al. 2011), that have been previously applied to gene network analysis (Pham et al. 2019, 2020, 2021), and used these to identify critical nodes. According to the network control method, any directed network can be controlled by a minimum set of nodes, called a minimum driver node set (MDNS) that can interact to control or drive the whole network. To identify the critical nodes within the gene regulatory network for each cancer type, the MDNS of the network is detected. To identify if a node is critical, that node will be removed from the original network, and a new MDNS will be calculated. If the MDNS increases, then the removed node is identified as a critical node.

### Collection of copper-related genes

To investigate cuproplasia-related prognostic effects for all downstream analyses in this study, we generated a 133-gene list. This gene list was obtained from copper-related gene sets in Molecular Signatures Database (MSigDB v2022.1.Hs) (Liberzon et al. 2015; Subramanian et al. 2005) as follows: WP_COPPER_HOMEOSTASIS, HP_DECREASED_CIRCULATING_COPPER_CONCENTRATION, HP_ABNORMAL_CIRCULATING_COPPER_CONCENTRATION, GOMF_COPPER_ION_TRANSMEMBRANE_TRANSPORTER_ACTIVITY, GOMF_COPPER_ION_BINDING, GOMF_COPPER_CHAPERONE_ACTIVITY, GOBP_RESPONSE_TO_COPPER_ION, GOBP_DETOXIFICATION_OF_COPPER_ION, GOBP_COPPER_ION_TRANSPORT, GOBP_COPPER_ION_TRANSMEMBRANE_TRANSPORT, GOBP_COPPER_ION_IMPORT, GOBP_COPPER_ION_HOMEOSTASIS, GOBP_CELLULAR_RESPONSE_TO_COPPER_ION, and GOBP_CELLULAR_COPPER_ION_HOMEOSTASIS. These gene sets were derived from different sources, including means of crowd sourcing, medical literature, Orphanet (a resource on rare diseases), DECIPHER (a database of genomic variation and phenotype in humans), and OMIM (a catalog of human genes and genetic disorders) (Supplementary Table 2 in Additional file 1). As these copper-related gene sets are related to abnormalities in human diseases, molecular functions, and biological processes, they may be appropriate to be used as candidates for investigating their clinical roles in tumours across cancer types. Then genes from each gene set were combined and overlapping genes are removed to obtain the final gene list (Supplementary Table 3 in Additional file 1).

### Univariate cox proportional hazards regression model

The R package ezcox (https://github.com/ShixiangWang/ezcox) was used to analyse the effects of copper-related genes on survival using the Univariate Cox proportional hazards regression model. Genes with *p*-value ≤ 0.05 were considered to have a statistically significant effect on survival and were selected for further analysis.

### Enrichment analysis

Enrichment analysis was conducted on the 30 critical cuproplasia-related genes identified in the pan-cancer analysis. Enrichr (Chen et al. 2013; Kuleshov et al. 2016; Xie et al. 2021) was used to identify the enriched gene sets from Gene Ontology Biological Processes and Molecular Function dataset and enrichment of pathways from Kyoto Encyclopedia of Genes and Genomes (KEGG) database.

### Survival analysis

To test if a cuproplasia-related gene signature infers survival, we used the SNFtool package (Wang et al. 2014) to compute the squared Euclidean distances between all pairs of patients using a vector formed with the gene expression values of that gene set. The patients were clustered into 2 groups based on their Euclidean distances to other patients using spectral clustering, identifying 2 subgroups of patients with the most similar expression profile of the gene set. Survival analysis was performed using the survival package (Therneau and Grambsch 2000) in R. This analysis was done for pan-cancer and individually for all 23 cancer types.

### Single nucleotide and copy number variant analysis

To explore the potential changed activity of copper-related genes in cancer development, we analysed the single nucleotide variants (SNVs) and copy number variations (CNVs) of the critical cuproplasia-related genes (CCGs). GSCALite (Liu et al. 2018a), a web server for analysing gene sets in cancer, was used for this exploration. This web server used the SNV and CNV data from the TCGA database. For the SNV data, we firstly selected only deleterious mutations, including Missense_Mutation, Nonsense_Mutation, Frame_Shift_Del, Splice_Site, Frame_Shift_Ins, In_Frame_Del, and In_Frame_Ins. We then kept mutations which had variant allele frequency larger than 0.1 and FILTER being PASS. Variant allele frequency was the ratio of read depth supporting the variant allele in tumour (i.e., t_alt_count) to read depth across this locus in tumour (i.e., t_depth). We obtained Revel scores of those mutations using Revel method (Ioannidis et al. 2016). Based on the Revel scores, the mutations were categorised into supporting benign, moderate benign, strong benign, supporting pathogenic, moderate pathogenic, and strong pathogenic. Only pathogenic mutations were retained for further analyses. We used maftools package (version 2.16.0) to show the mutation distribution and type of SNVs for genes in an oncoplot. CNV data was already annotated as homozygous deletion, heterozygous deletion, heterozygous amplification or homozygous amplification within GSCALite. The homozygous deletion and amplification CNV events were combined with the pathogenic SNVs to separate patients into either mutant or wild type (WT). Survival analysis was performed using the survival package in R. This analysis was done pan-cancer for 29 CCGs (*MT-CO1* was excluded due to missing data).

### Construction and validation for a risk score model in low grade glioma (LGG)

A risk score model was constructed using the Least Absolute Shrinkage and Selection Operator (LASSO) regression model from glmnet package (Friedman et al. 2010) using gene expression of the CCGs in LGG and survival data. Univariate Cox regression model was firstly used for the 13 CCGs of LGG to select 10 genes which were individually related to survival, then LASSO was applied to these 10 genes. Cross-validation confirmed the optimal model performance at log(λ) = -3.613 for 6 genes, implying that these 6 genes can be used to build the risk score model. The model was built using the gene expression data (expected counts from RSEM output) from the TCGA database (i.e., training set). To validate the risk score model we obtained gene expression and survival data from LGG patients in the CGGA database (http://www.cgga.org.cn/) (Liu et al. 2018b; Wang et al. 2015; Zhao et al. 2021) (i.e., test set). The receiver operating characteristic (ROC) curve was plotted and the area under the ROC curve (AUC) was computed using the R package timeROC (Blanche et al. 2013).

## Results

### A gene regulatory network identifies critical cuproplasia-related genes in pan-cancer

**Fig. 1:**
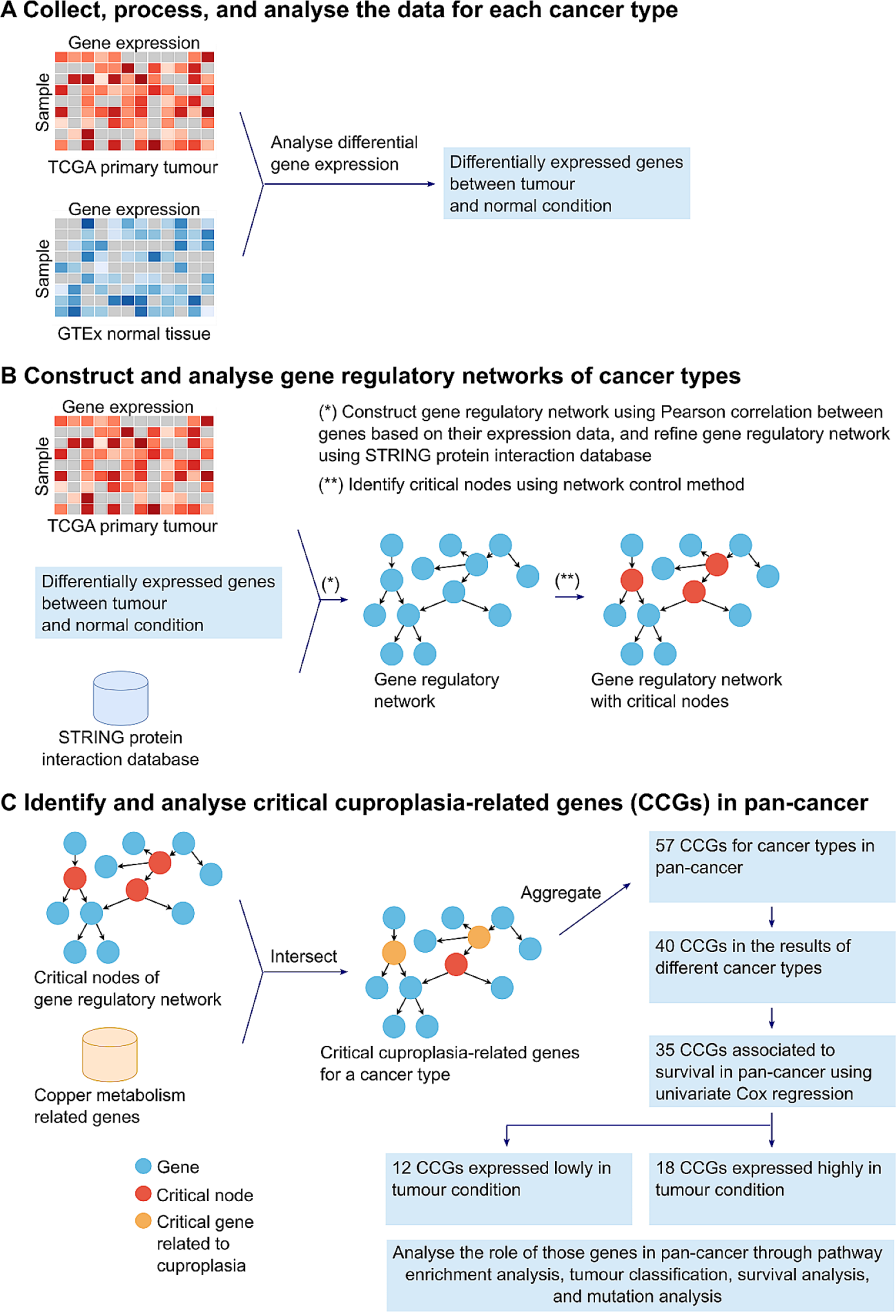
Gene regulatory network methodology to identify 30 critical cuproplasia-related genes pan-cancer (**A**) Collect, process, and analyse the gene expression data of TCGA primary tumours and GTEx normal tissues to identify differentially expressed genes between tumour and normal tissue for each cancer type. (**B**) Construction of gene regulatory networks for each cancer type using the differentially expressed genes and STRING protein interaction database. The network control method was used to indicate critical nodes for each cancer type. (**C**) Identification of critical cuproplasia-related genes from the gene regulatory network that identified 12 low and 18 high expressed genes in tumour using a pan-cancer approach

Gene regulatory networks can be used to elucidate molecular mechanisms in disease progression (Liu et al. 2016; Yu et al. 2017). To identify if there are CCGs that impact patient survival, we sought to generate a gene regulatory network to identify CCGs. We obtained gene expression data from 23 tumours and normal tissue samples from TCGA and GTEx respectively. Differential gene expression analysis identified for each cancer type genes that were significantly up- or down-regulated in tumour samples (Fig. 1A; Supplementary Table 4 in Additional file 1). An individual gene regulatory network was built (see methods) for the 23 cancer types (Fig. 1B; Table 1). To further identify the CCGs, we intersected the critical gene nodes in the estimated networks for each cancer type with copper metabolism related genes (113-gene set; Supplementary Table 3 in Additional file 1). These critical genes are identified as they play a central role in controlling the gene networks and resultant expression alterations in these genes may transform the state of a cell from normal to pre-cancerous or malignant (Table 2).

**Table 1:** Gene regulatory network for each cancer type specifying the number of nodes, edges and critical nodes that make up the gene regulatory network

Cancer type	Number of nodes	Number of edges	Number of critical nodes
ACC	2257	11,361	185
AML	4935	23,601	503
BLCA	3300	21,205	307
BRCA	4387	25,612	432
COAD	5736	47,240	581
ESCA	4177	21,570	414
GBM	5879	44,964	601
KICH	5193	26,799	522
KIRC	5057	33,674	515
KIRP	4600	25,811	474
LGG	4417	25,600	461
LIHC	2537	14,324	243
LUAD	4100	21,414	403
LUSC	5789	44,649	549
OVCA	6612	47,400	645
PAAD	4893	30,320	510
PCPG	3840	11,691	400
PRAD	3771	18,033	371
SKCM	4546	23,085	493
STAD	4077	23,764	416
THCA	3413	17,283	346
UCEC	5721	36,833	582
UCS	3492	12,502	286

**Table 2:** Critical cuproplasia-related genes identified from the gene regulatory network for each cancer type

Cancer type	Number of CCGs	CCGs
ACC	3	*CDK1, COX17, DBH*
AML	12	*SLC11A2, MAP1LC3A, ALB, CDK1, ANG, ANKRD9, AP1S1, ARF1, APC, TMPRSS6, GPC1, LOXL1*
BLCA	4	*CDK1, ATOX1, MAPT, COX17*
BRCA	13	*ALB, ARF1, CASP3, XIAP, GSK3B, AOC3, S100A12, CDK1, FOXO1, JUN, MAP1LC3A, SNCA, SORD*
COAD	10	*AP1S1, ALB, APP, CASP3, AOC3, CDK1, PRNP, SNCA, ATP6AP1, F5*
ESCA	9	*ADAM10, ADAM17, GSK3B, CDK1, MAP1LC3A, MAPT, COA6, MT-CO2, STEAP3*
GBM	10	*ALB, CYP1A1, CASP3, ANG, SNCA, MAPT, FOXO1, JUN, CP, MMGT1*
KICH	7	*ALB, BACE1, CASP3, AP1B1, CDK1, TMPRSS6, FOXO1*
KIRC	10	*XIAP, APP, GSK3B, CYP1A1, S100A12, PRNP, JUN, TP53, ATP7A, SP1*
KIRP	8	*TMPRSS6, ALB, XIAP, APP, AP1S1, CASP3, GSK3B, CCND1*
LGG	13	*XIAP, ALB, CASP3, SNCA, CDK1, ATP7A, MT-CO1, CP, CYP1A1, F5, MAP1LC3A, SP1, TP53*
LIHC	4	*CDK1, AP1S1, COA6, PRND*
LUAD	8	*AANAT, CYP1A1, CDK1, DBH, AP1S1, AQP1, MT1X, TP53*
LUSC	8	*GSK3B, CYP1A1, MAP1LC3A, JUN, IL1A, SPATA5, SORD, TP53*
OVCA	13	*AP1S1, TMPRSS6, ALB, CASP3, GSK3B, CDK1, SNCA, MT-CO2, COA6, COMMD1, COX17, MT-CO1, SLC11A2*
PAAD	11	*TMPRSS6, ADAM10, APP, BACE1, ALB, CDK1, CASP3, GPC1, F5, F8, SUMF1*
PCPG	10	*AP1S1, AOC3, APP, MAPT, SNCA, CDK1, ATP7A, MAP1LC3A, COX17, GPC1*
PRAD	7	*ALB, ARF1, APP, AP1S1, CDK1, MAPT, XAF1*
SKCM	10	*TMPRSS6, ALB, CDK1, SNCA, MAPT, JUN, MAP1LC3A, HEPH, IL1A, MT1X*
STAD	6	*ALB, CDK1, AP1S1, JUN, SP1, SORD*
THCA	10	*MAP1LC3A, XIAP, CYP1A1, ALB, CDK1, ATP6AP1, COA6, GSK3B, MT-CO1, TMPRSS6*
UCEC	12	*AP1S1, CASP3, CDK1, AQP1, PARK7, MAPT, XAF1, PRNP, COX17, TP53, SNCA, PRND*
UCS	3	*AP1S1, FOXO1, LCAT*

**Fig. 2:**
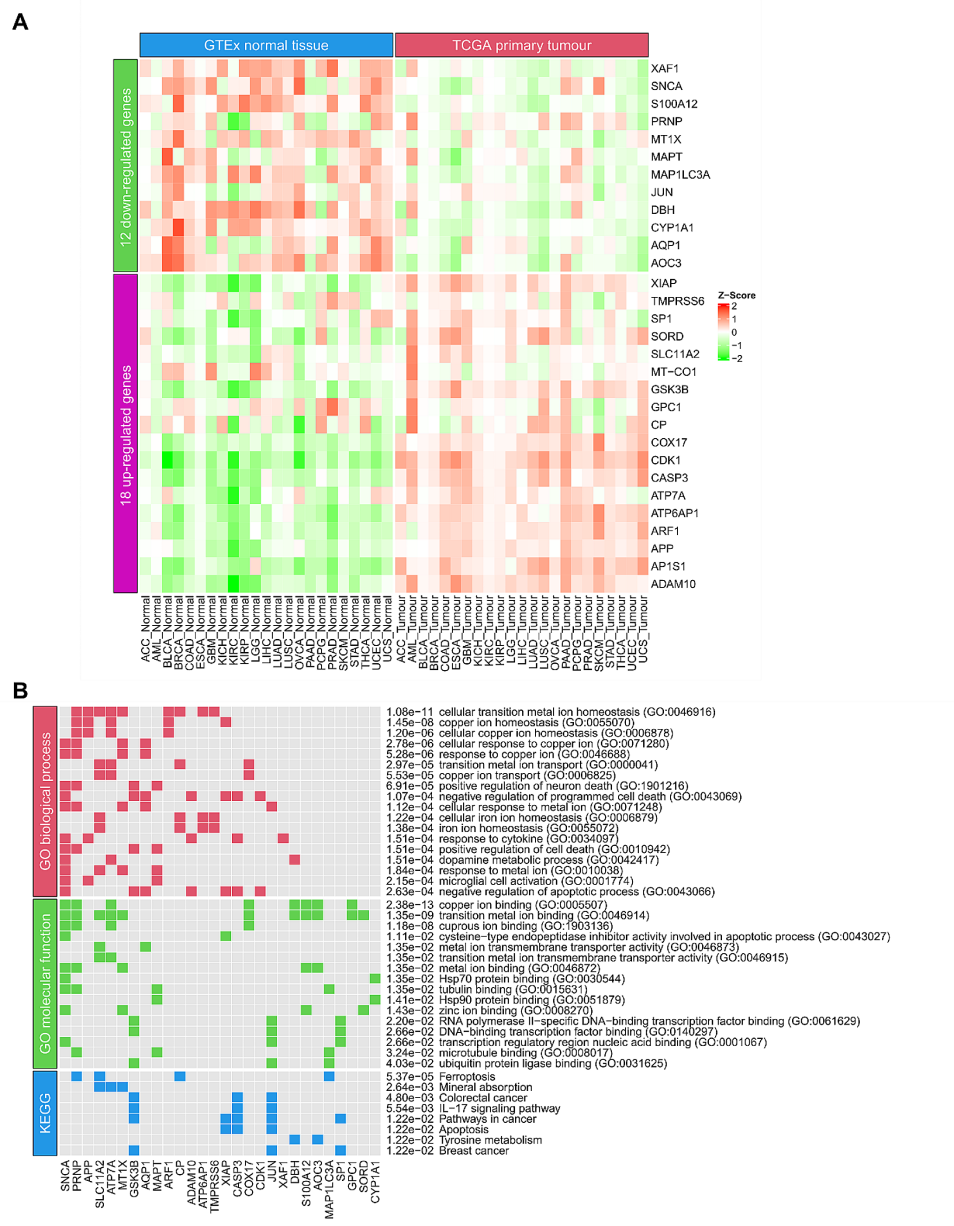
Critical cuproplasia-related genes are differentially expressed pan-cancer (**A**) Heatmap of the 18 up-regulated and 12 down-regulated genes in primary tumours compared to corresponding normal tissue in the 23 cancer types. The mean gene expression values in each primary tumour and corresponding normal tissue are used to generate the heatmap. (**B**) The top significantly enriched gene ontology (GO) Biological Processes (top; red), Molecular Function (middle; green) and KEGG pathways (bottom; blue) from the genes identified in A ordered by their adjusted *p*-values on the right. Coloured cells indicate that a critical cuproplasia-related gene is enriched in the corresponding term

Next, we applied this method to each cancer type and identified the critical cuproplasia-related genes in pan-cancer. We aggregated all the identified CCGs in the 23 cancer types into a single set and removed duplicated genes, resulting in 57 genes (Supplementary Table 5 in Additional file 1, Fig. 1C). Of these 40 genes occurred in multiple cancer types (Fig. 1C). From these genes, we identified *ATOX1*, which is known to play a functional role as antioxidant against superoxide and hydrogen peroxide (O’Leary et al. 2016) and may play a significant role in cancer carcinogenesis (O’Leary et al. 2016). Furthermore, we identified *CCND1*, which encodes the protein belonging to the highly conserved cyclin family. Notably, mutations and overexpression of *CCND1* alter cell cycle progression and are observed frequently in a variety of human cancers (O’Leary et al. 2016). In addition, *CYP1A1* encodes a member of the cytochrome P450 superfamily of enzymes and is associated with lung cancer (O’Leary et al. 2016). *CYP1A1* is identified as a CCG in both lung adenocarcinoma (LUAD) and lung squamous cell carcinoma (LUSC), reaffirming our methodology to identify CCGs. We sought to further refine the list of CCGs to identify a potential specific subset of genes whose expression may be prognostic of pan-cancer survival. Univariate Cox proportional hazards regression model based on the 40 genes revealed that 35 genes were significantly associated with survival (*p* < 0.05; Fig. 1C; Supplementary Table 5 in Additional file 1). Of these 18 genes were up-regulated and 12 down-regulated in tumours compared to matched normal tissue expression (Figs. 1C and 2A; Supplementary Table 5 in Additional file 1). A gene was considered as up-regulated/ down-regulated if it was significantly up-regulated/ down-regulated in most cancer types for which it was identified as a CCG. Unsurprisingly, enrichment analysis showed that the 30 CCGs were involved in biological processes and molecular function related to copper homeostasis, binding and cellular response to copper ions (Fig. 2B). Furthermore, pathway enrichment confirmed the relationship with these genes appearing in pathways in cancer and importantly significantly associated with colorectal and breast cancer (Fig. 2B). These findings confirm the association between the identified CCGs to the progression of cancer and cuproplasia in cancer. Importantly, these results confirm that utilising gene regulatory networks is a powerful methodology to identify novel gene-sets impacting disease.

### Cuproplasia-related gene signature predicts patient survival

**Fig. 3:**
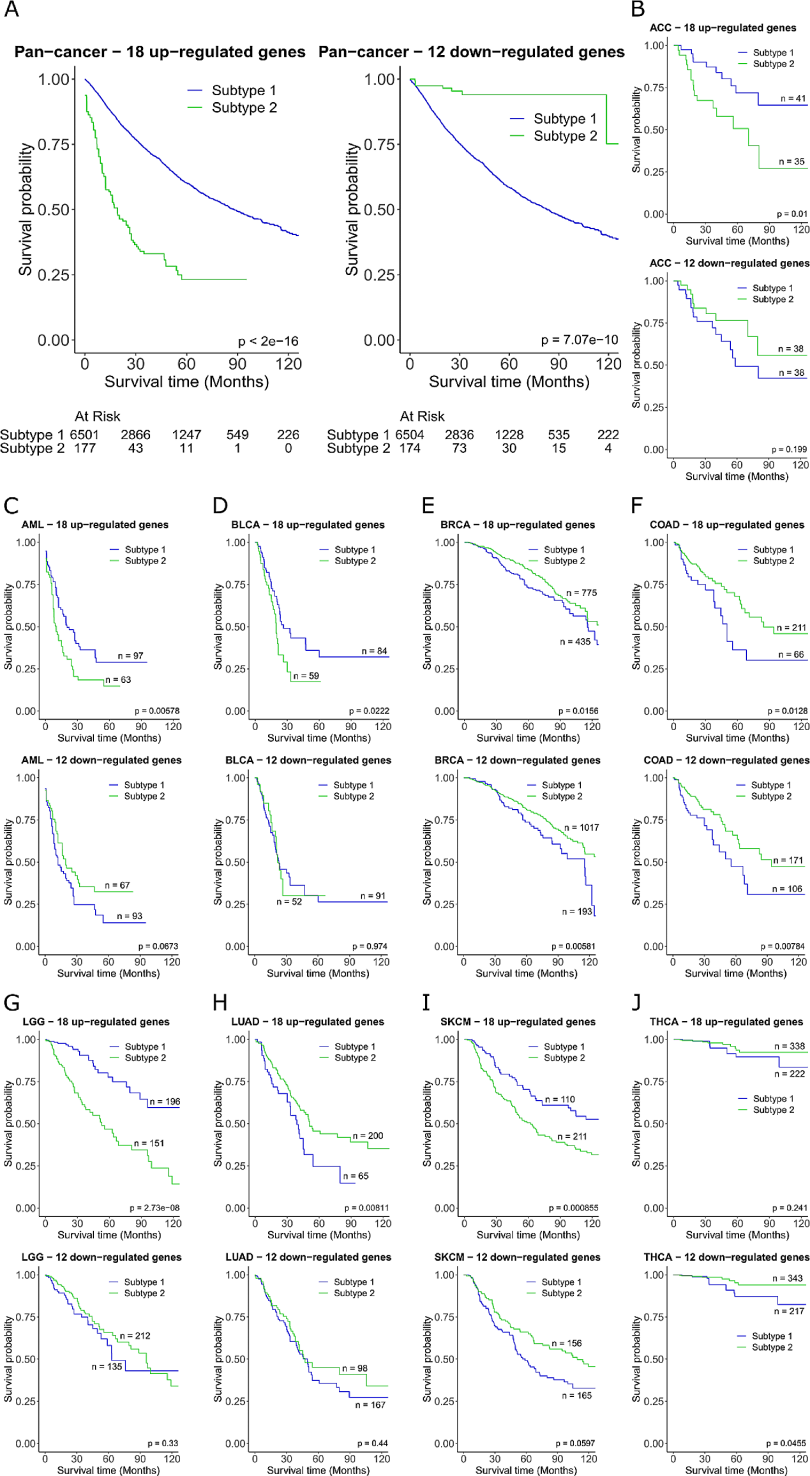
Cuproplasia-related gene signature predicts survival Survival analysis for the 18 up-regulated and 12 down-regulated CCGs (**A**) pan-cancer (*N* = 6678), (**B**) ACC (*N* = 76), (**C**) AML (*N* = 160), (**D**) BLCA (*N* = 143), (**E**) BRCA (*N* = 1210), (**F**) COAD (*N* = 277), (**G**) LGG (*N* = 347), (**H**) LUAD (*N* = 265), (**I**) SKCM (*N* = 321), and (**J**) THCA (*N* = 560)

From the gene regulatory network, we created to identify the CCGs, we wanted to determine if the 30 genes could be utilised as potential biomarkers to predict tumour prognosis. To explore this concept further, the 18 up-regulated and 12 down-regulated genes described above (Fig. 2A) were used separately to stratify pan-cancer patients to see if tumour expression profiles inferred a better prognostic outcome. We analysed the 18 up-regulated genes and 12 down-regulated genes independently. Euclidean distance of patients based on the gene expression signature was used for spectral clustering to dichotomise patients into subtype 1 and subtype 2, with the patients in each subtype having similar expression profiles of the gene set. A significant decrease in survival (*P* < 2e-16) was observed in subtype 2 compared to subtype 1 using the up-regulated genes, and significant increase in survival (*P* = 7.07e-10) using the down-regulated genes (Fig. 3A). The same approach was then applied individually to the 23 cancer types (Fig. 3B-J, Supplementary Fig. 1 in Additional file 2). Cancer type specific survival analysis was only significant for the 18 gene up-regulated signature in ACC, AML, BLCA, BRCA, COAD, LGG, LUAD, and SKCM, and significant in the 12 gene down-regulated signature in BRCA, COAD, and THCA (Fig. 3B-J). Taken together this shows that the identified gene signatures are only predictive of survival for a subset of cancer types even though the signature can be applied in pan-cancer. However, these results indicate that CCG-related signatures offer a novel tumour classification method in cancer patients, with prognostic power and have the potential to be used as biomarkers.

**Fig. 4:**
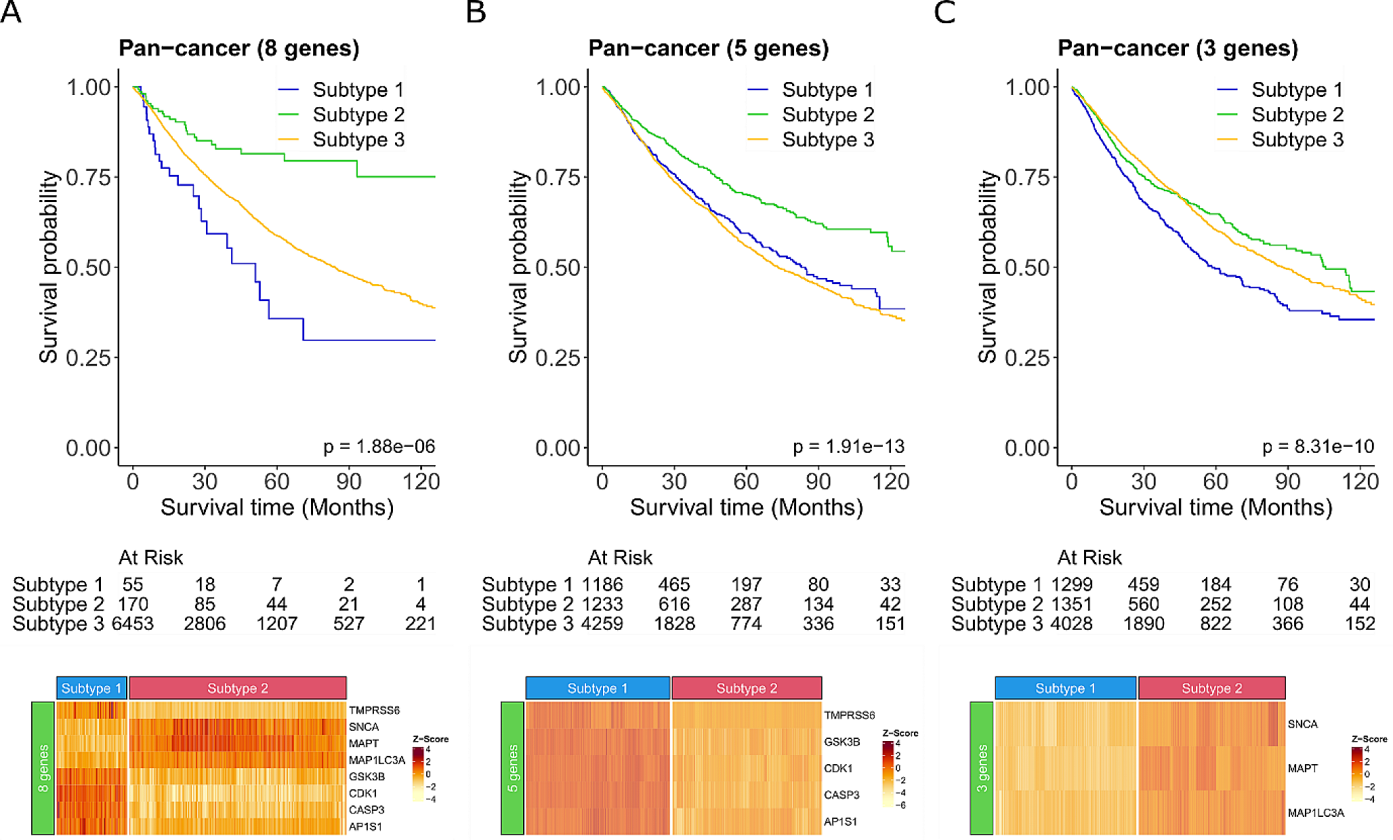
A novel cuproplasia-related 8-gene signature is a potential biomarker of improved survival Survival curves (top) and gene expression heatmaps (bottom) for all patients for: (**A**) 8-gene signature consisting of *CDK1, AP1S1, CASP3, MAP1LC3A, SNCA, TMPRSS6, MAPT*, and *GSK3B.* Subtype 1 are patients with low expression of *MAP1LC3A*, *SNCA*, and *MAPT* and high expression of *CDK1*, *AP1S1*, *CASP3*, *TMPRSS6*, and *GSK3B*. Subtype 2 are patients with high expression *MAP1LC3A*, *SNCA*, and *MAPT* and low expression of *CDK1*, *AP1S1*, *CASP3*, *TMPRSS6*, and *GSK3B.* Subtype 3 are all other patients. (**B**) The 5 up-regulated genes comparing primary tumour with normal tissue. Subtype 1 are patients with high expression and subtype 2 low expression in *CDK1*, *AP1S1*, *CASP3*, *TMPRSS6*, and *GSK3B.* Subtype 3 are all other patients. (**C**) The 3 down-regulated genes comparing primary tumour with normal tissue. Subtype 1 are patients with low expression and subtype 2 with high expression in *MAP1LC3A*, *SNCA*, and *MAPT.* Subtype 3 are all other patients. Heatmaps in A-C are only shown for patients in subtype 1 and 2

Our initial approach dichotomised patients into two subgroups dependent on the similarity of either the 18 up-regulated or 12 down-regulated CCG-related signatures to infer survival. However, the expression profiles of the CCG-related genes for the two subgroups of patients were not consistent with the expected directionality of gene expression level (Supplementary Fig. 2 in Additional file 2). Therefore, to further understand the relationship of the 30 CCGs identified and survival for use as a potential prognostic biomarker, we needed to further refine the signature to identify those genes that are consistent with the primary tumour expression. We combined the 18 up-regulated and 12 down-regulated genes to identify the specific expression dependent gene combination that most contributes to survival pan-cancer. Tumours were annotated for each of the 30 genes as having either high or low expression using the median expression value across the cohort as the threshold. This resulted in a refined 8 gene pan-cancer specific signature that significantly improved survival (*P* = 1.88e-06; Fig. 4A). Patients whose tumour had high expression of *MAP1LC3A*, *SNCA*, and *MAPT* and low expression of *CDK1*, *AP1S1*, *CASP3*, *TMPRSS6*, and *GSK3B* inferred better overall survival than all other patients (Fig. 4A). Conversely patients who had low expression of *MAP1LC3A*, *SNCA*, and *MAPT* and high expression of *CDK1*, *AP1S1*, *CASP3*, *TMPRSS6*, and *GSK3B*, had the poorest overall survival. Further separating the genes into the 5 highly expressed and 3 lowly expressed genes also showed a significant improvement in survival (*P* = 1.91e-13 and *P* = 8.31e-10, respectively), however, resulted in poorer survival probability than the combined 8 gene signature (Fig. 4A-C). Based on these findings it is the combination of the 3 down-regulated and 5 up-regulated genes together that make up this novel signature that is more predictive of survival. Thus, our novel 8-gene CCGs could potentially be used to stratify patients prognostically regardless of cancer type. Additionally, we also used sigQC (Dhawan et al. 2019) to systematically evaluate this 8-gene signature (Supplementary Fig. 3 in Additional File 2). The results showed the compactness (i.e., low intra-signature correlation), large proportion of samples with high expression, and variability of the signature genes, indicating the high quality of the gene signature. With the low scoring metrics based on PCA1, mean, and median of the expression of the signature genes, we may not summarise the signature as a single score.

### Mutations in critical cuproplasia-related genes are associated with poorer survival

**Fig. 5:**
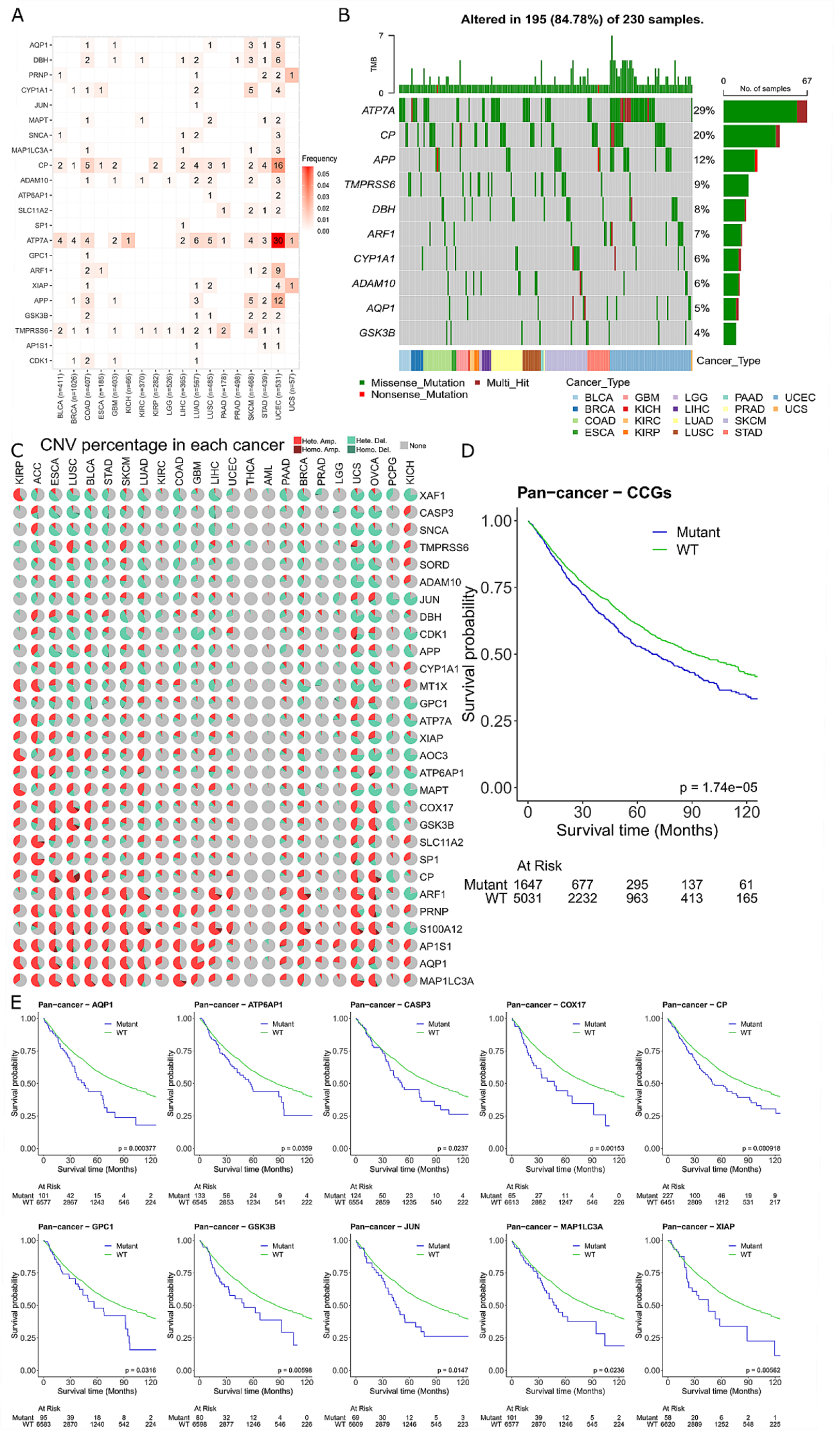
Mutations in CCGs have a poorer survival for cancer patients (**A**) Number of pathogenic single nucleotide variants (SNVs) for the 22 CCGs across 18 cancer types from the TCGA database. Cells are highlighted based on the frequency of the number of patients with the specified gene mutation in the given cancer. The darker reads the higher the frequency. The total number of patients in a specific cancer type is shown on the x-axis (i.e., the n in parentheses). (**B**) An oncoplot ordered by cancer type, showing the mutation distribution and type of SNVs for the top 10 most frequently mutated genes. A gene was classified as a multi-hit (Multi_Hit; dark red) if a patient had more than one mutation in the given gene. Top annotation shows the number of mutations in each patient. Right annotation is the number corresponding to the different types of mutations. Bottom annotation is the cancer type. (**C**) Pie charts summarising the percentage of patients with copy number variations (CNVs) in the 29 CCGs (*MT-CO1* was removed due to the missing data) across 23 cancer types from the TCGA database. CNVs were classified into four types: heterozygous amplification (Hete. Amp), homozygous amplification (Homo. Amp.), heterozygous deletion (Hete. Del.), and homozygous deletion (Homo. Del.). (**D**) Survival analysis for mutant and WT patients pan-cancer (*N* = 6678). (**E**) Survival analysis for mutant and WT patients for the 10 genes (*AQP1*, *ATP6AP1*, *CASP3*, *COX17*, *CP*, *GPC1*, *GSK3B*, *JUN*, *MAP1LC3A*, and *XIAP*) with significant overall survival pan-cancer

Next, we wanted to observe the impact of mutations on the identified 30 CCGs. Using the TCGA mutation data for the 30 genes we extracted the SNVs and the CNVs for each of the patients across all the cancer types. SNVs were further filtered (see methods) to identify only those mutations that have previously been annotated as pathogenic and thus more likely to be deleterious. A pathogenic mutation was observed in 22 of the CCGs, and only present in 18 of the 23 cancer types analysed (Fig. 5A). Mutations in *ATP7A* and *CP* occurred the most frequently, with the highest number of pathogenic mutations occurring in UCEC (Fig. 5A). Mutations were exclusively either missense or nonsense mutations, with some patient samples having more than one SNV in the same gene (Fig. 5B). The mutations predominantly occurred within protein domains and for the top 10 most highly mutated genes, there was no single mutation present but rather span the protein coding region of the entire gene (Supplementary Fig. 4 in Additional file 2). Expanding mutation analysis to include CNVs within the 29 CCGs (*MT-CO1* was excluded due to missing data), there was a degree of copy number change observed across all cancer types (Fig. 5C). *MAP1LC3A*, *AQP1*, and *AP1S1* were consistently amplified across cancer types whilst *XAF1*, *CASP3*, and *SNCA* exhibited copy deletions.

We combined the identified pathogenic SNV and homozygous deletions and amplifications of CNV data together to further understand how the presence of a mutation in the CCGs impacts patient survival. Patients were classified as either mutant or wild type (WT) for at least one of the 30 CCGs and revealed that those patients with a mutation had significantly poorer overall survival (*P* = 1.74e-05; Fig. 5D). We further separated the survival analysis into individual genes to understand if poorer survival was influenced by a specific gene. We found that 10 genes were significantly associated with poorer survival when a mutation was present (Fig. 5E) and had no effect on survival in 19 genes (Supplementary Fig. 5 in Additional file 2) (*MT-CO1* was excluded due to missing data). Of the 10 significant genes associated with survival, it is important to note that only 3 of these genes (*MAP1LC3A*, *GSK3B* and *CASP3*) were identified in the novel 8-gene signature we identified previously (Fig. 4). Taken together this shows the utility of utilising a gene regulatory network to identify CCGs that are associated with survival pan-cancer, and a comprehensive genomic analysis of both mutations and expression is required.

### Critical cuproplasia-related genes identify a prognostic risk model for low Grade Glioma

**Fig. 6:**
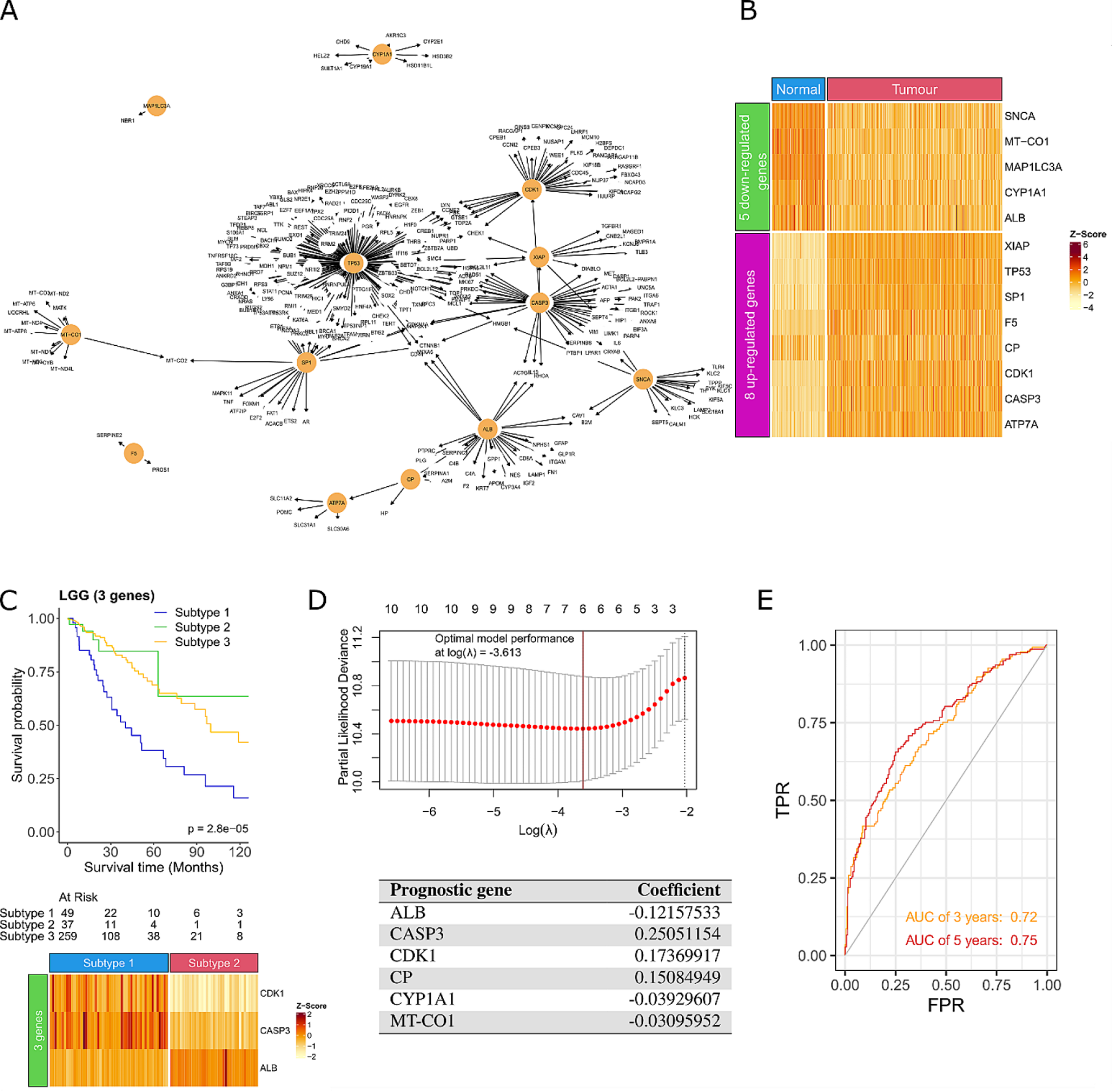
Critical cuproplasia-related genes in Low Grade Glioma (**A**) Gene regulatory network of the 13 CCGs in LGG. Nodes are genes and edges are the interactions between the nodes. (**B**) Heatmap of the 8 up-regulated and 5 down-regulated genes in LGGs (*N* = 348) compared to normal brain tissue (*N* = 105). (**C**) Survival curve (top) and gene expression heatmap (bottom) for the 3-gene signature most predictive of survival. Subtype 1 patients have high expression of *CDK1* and *CASP3* and low expression of *ALB*. Subtype 2 patients have low expression of *CDK1* and *CASP3* and high expression of *ALB*. Subtype 3 are all other patients. Three patients are removed due to missing survival data (i.e., there are 345 patients left for this analysis). (**D**) The optimal model performance with the minimum partial likelihood deviance on the y-axis for the risk score model tuning for the lambda parameter (log(λ) = -3.613) (top) for the 6 prognostic genes (bottom) with the corresponding coefficients in the risk score model. (**E**) Validation dataset obtained from CGGA for the risk score model with area under the ROC curve (AUC) for 3- and 5-year survival

As we showed previously (Fig. 3) whilst the pan-cancer survival analysis of the 30 CCGs showed improved survival, it was only predictive in a subset of cancer types. Therefore, we wanted to apply the same gene regulatory network method developed in Fig. 1 and focus solely on low grade glioma (LGG). We chose to explore LGG as the survival difference of LGG patient subgroups clustered using the 18 up-regulated CCGs was significant (*P* = 2.73e-8; Fig. 3G) and it is a cancer type already recognised to be impacted by intracellular copper levels (Chen et al. 2022). The LGG gene regulatory network identified an initial 4,417 nodes and 25,600 edges, of which 461 were identified as critical nodes in the network. Intersecting the critical nodes with the 133-copper metabolism related genes (Supplementary Table 3 in Additional file 1) identified a network consisting of 13 CCGs (Fig. 6A). Of these, 8 genes were up-regulated (*XIAP*, *TP53*, *SP1*, *F5*, *CP*, *CDK1*, *CASP3*, and *ATP7A*) and 5 genes were down-regulated (*SNCA*, *MT-CO1*, *MAP1LC3A*, *CYP1A1*, and *ALB*) in LGG samples compared to matched normal brain tissue (Fig. 6B). Interestingly, *CASP3* and *CYP1A1* were previously identified to be related to glioma and used to predict the survival for glioma patients (Chao et al. 2022; Liu et al. 2022b), the high expression of *CDK1* is associated with the malignant progression in glioma (Chen et al. 2007; Teng et al. 2022), and mutations in *MT-CO1* contribute to brain tumours (Kaneva et al. 2021). We further refined this list to identify a LGG specific biomarker signature that is predictive of response. A 3-gene signature (2 up-regulated *CDK1*, *CASP3* and 1 down-regulated *ALB*) was identified, and patients who had high expression of *ALB* and low expression of *CDK1* and *CASP3* had significantly better survival (*P* = 2.8e-05; Fig. 6C). We also verified this signature on another dataset (i.e., CGGA) (Supplementary Fig. 6 in Additional file 2). Interestingly, we discovered that the signature worked in the expected manner (i.e., the signature could be used to classify LGG patients into different groups with significant difference in survival). Next, we wanted to see if we could generate a risk prediction model from the 13 CCGs to predict survival in LGG patients. Univariate Cox regression was applied to the 13 genes and identified 10 genes as potential risk factors related to survival (*P* < 0.05; Supplementary Table 6 in Additional file 1). LASSO regression was then applied to the 10 significant genes which identified *ALB, CASP3*, *CDK1*, *MT-CO1*, *CP*, and *CYP1A1* as prognostic (Fig. 6D). The optimal model performance was achieved with these 6 genes, implying that these genes can be used to build the risk score model (Fig. 6D). We validated the risk score model on another dataset from the Chinese Glioma Genome Atlas (CGGA), resulting in an area under the curve value of 0.72 after 3 years and 0.75 after 5 years (Fig. 6E). Importantly, the 3-gene signature identified are also prognostic in the risk score model (*CDK1*, *CASP3* and *ALB*). Taken together, the validation dataset provides the supporting evidence that the 6 identified CCGs in LGG has the potential to be a prognostic biomarker for LGG patients.

## Discussion

In this study, a novel framework based on gene regulatory networks was proposed to evaluate cuproplasia-related genes across 23 cancer types. Utilising publicly available data from TCGA primary tumours, we constructed a gene regulatory network. This identified 30 CCGs that were enriched in pathways involved in cancer progression, immune evasion and apoptosis. As the identified CCGs play critical roles in cancer development, these genes could be used as potential biomarkers for tumour classification. Therefore, survival analysis identified a refined novel 8-gene signature significantly associated with prognosis. As copper is a well-known micronutrient which is essential for the metabolism of cancer cells and the role of copper in LGG has been previously reported (Bao et al. 2022), we applied our methodology to LGG and identified a risk score model consisting of 6 cuproplasia-related genes to be predictive of survival. Understanding the role of copper and cuproplasia in cancer development is important for selecting patients who can benefit from copper chelation therapy and designing novel and effective combination treatments to reduce drug resistance. We can then utilise this knowledge to design suitable treatment plans for patients to maximise their treatment outcomes.

The novel 8-gene signature we identified consisted of *CDK1*, *AP1S1*, *CASP3*, *MAP1LC3A*, *SNCA*, *TMPRSS6*, *MAPT*, and *GSK3B*. Although these genes are known for their important roles in various malignancies and are associated with poor survival (Guven et al. 2021; Huang et al. 2011; Liu et al. 2022a; Mete et al. 2020; Sofi et al. 2022; Zheng et al. 2022; Zhou et al. 2022), this is the first paper indicating their connection with the dysregulation of copper levels in cancer patients. It is important to highlight that our identified CCGs have been confirmed to be involved in a wide range of biological processes supporting tumour progression and immune evasion processes. The specific processes related to the identified CCGs include autophagy, cell cycle regulation, cell proliferation, kinase signalling pathways, immune infiltration and immune checkpoint regulation.

Copper has been previously demonstrated as an essential regulator of autophagy, specifically regulating autophagic kinase activity. This self-degradation process in which the cell recycles cytoplasmic constituents has been previously linked to cancer formation and progression. Here, we further strengthen the link between copper and autophagy with our gene-signature highlighting *MAP1L3CA, CDK1, CASP3* and *SNCA*, all of which possess involvement in autophagic processes. Importantly, *SNCA* has also been shown to play a potential role in pathological processes in lung adenocarcinoma (Zhang et al. 2022), ovarian cancer (Bruening et al. 2000), breast cancer (Bruening et al. 2000), colorectal tumour (Ye et al. 2010), melanoma (Turriani et al. 2017), and brain cancer (Kawashima et al. 2000), highlighting the strength of this gene-signature across distinct tumour types. Kinase signalling pathways play a large role in nearly all cellular processes, and copper has a documented strong influence on these pathways. Our gene-signature also reflects this influence, with *CDK1* dysregulation a hallmark of cell cycle dysregulation, and *GSK3B* dysregulation observed in homeostatic dysfunction, both of which constitute key hallmarks of malignancies. In particular, the abnormal expression and activity of *GSK3B* has been demonstrated to influence metabolic and immune evasion signalling. Specifically, recent investigations demonstrated that activation of *AKT* by *EGFR* suppresses *GSK3B* activity through Ser9 phosphorylation and this induced *PD-L1* destabilization (Li et al. 2016). Consistently, we previously demonstrated that copper chelation therapy inhibited *GSK3B* phosphorylation and in turn induced downregulation of *PD-L1* and increased anti-cancer immune response (Rouaen et al. 2022; Voli et al. 2020). In regards to immune processes, high expression of *AP1S1* in tumours was negatively correlated with immune infiltrating cells (Zheng et al. 2022), and low expression of *SNCA* was correlated to low levels of immune infiltration (Zhang et al. 2022), indicating that these genes play a key role in the anti-cancer immune response and immune evasion mechanism. *AP1S1* also plays a crucial role in clathrin coat assembly and mediates trafficking between the trans-Golgi network, endosomes and the plasma membrane. *AP1S1* has an important role in regulating copper accumulation and homeostasis in cells (Martinelli et al. 2013). *CASP3* is a key mediator of apoptosis during cellular exposure to cytotoxic drugs, radiotherapy, or immunotherapy (Zhou et al. 2018). Given the important roles of these genes in tumour formation and development, the use of therapeutic strategies targeting copper could have a strong effect on these genes and improve the prognosis of patients affected by different types of tumours.

According to our findings, cancer patients with low expression of *MAP1LC3A, SNCA*, and *MAPT* and high expression of *CDK1, AP1S1, CASP3, TMPRSS6*, and *GSK3B* have poor overall survival while patients with high expression of *MAP1LC3A, SNCA*, and *MAPT* and low expression of *CDK1, AP1S1, CASP3, TMPRSS6*, and *GSK3B* have good overall survival. Thus, we may improve the survival chance of patients with low expression of *MAP1LC3A, SNCA*, and *MAPT* and high expression of *CDK1, AP1S1, CASP3, TMPRSS6*, and *GSK3B* if we can control the expression of these genes.

Copper chelation therapy was developed to treat Wilson’s disease which is a genetic disease with an excess accumulation of copper in liver and brain. In our previous studies, we discovered that copper chelation therapy may result in regulating the expression of genes. For example, copper chelation downregulates transcriptomic expression of EMT markers and transcription factors in cancer cell lines (Poursani et al. 2023), or copper chelation therapy inhibited GSK3B phosphorylation and in turn induced downregulation of PD-L1 and increased anti-cancer immune response (Rouaen et al. 2022; Voli et al. 2020). Thus, we may use copper chelation therapy and/or other therapies to target the signature genes to regulate their expression in cancer patients. As a result, by using our signature we can select patients to be treated with copper chelation therapy to maximise their potential beneficial effects in term of improved survival.

Our study proposed a novel network-based method for identifying cuproplasia-related gene signatures for cancer patients, and our main analyses were for pan-cancer. However, since cancer types have different morphologies and clinical outcomes, they might have different causes and gene signatures. Additionally, our method can be applied to other datasets or a specific cancer type. In particular, we have chosen LGG as it is a diverse group of primary brain tumours that often arise in young patients and generally have an indolent course with longer-term survival in comparison with high-grade gliomas and other aggressive cancer types. Thus, besides the pan-cancer signature, we also applied our method to the data of LGG patients for identifying a gene signature which was for them, illustrating the capability and flexibility of our proposed method in the application.

As a result, the pan-cancer signature included 8 genes *CDK1, AP1S1, CASP3, MAP1LC3A, SNCA, TMPRSS6, MAPT*, and *GSK3B*, and the LGG signature included 3 genes *CDK1, CASP3*, and *ALB*. It is understandable that the pan-cancer signature does not include all the genes of the LGG signature, because some genes may play critical roles in a specific cancer type such as LGG but in the context of pan-cancer, they may not be as important as other genes. However, as you can see in the results there are two genes (i.e., *CDK1* and *CASP3*) which are in the signatures of both pan-cancer and LGG, indicating that these two genes are important not only in LGG but also in other cancer types.

Since our proposed method used the gene expression data of patients from 23 cancer types, the identified pan-cancer signature included the information of these cancer types. However, due to the imbalance of the number of patients among the cancer types, a potential bias might occur in the results. We examined this issue by reducing the prevalence of breast cancer samples (i.e., we only used a half number of breast cancer patients, including 606/1212 randomly selected patients), and we identified a new signature including 7 genes *CDK1, AP1S1, CASP3, MAP1LC3A, SNCA, MAPT*, and *GSK3B* (Supplementary Fig. 7 in Additional file 2). In general, this signature was similar to the previous one, and these 7 genes were included in the 8-gene signature. Furthermore, the new signature can also be used to cluster patients into different groups with significant difference in survival. Thus, although the potential bias might be not significant with the prevalence of samples in some cancer type, to minimise the bias when using our proposed method, we may need to consider having a balance of the number of samples among cancer types.

Another limitation of our proposed method is that we used the STRING protein interaction database to construct gene regulatory networks for each of the 23 cancer types. We wanted to include only interactions which occurred between proteins as they were the most translatable interactions to pathway signalling. However, this selection might eliminate potential interactions that may occur such as RNA-RNA interactions since not all RNAs is translated to protein expression. As a future work for improving our proposed method, instead of using the STRING protein interactions only we may consider including both protein-protein interactions and RNA-RNA interactions for building gene regulatory networks.

## Conclusions

In this paper, we conducted for the first time a comprehensive pan-cancer analysis for genes involved in cuproplasia using a novel framework. An 8-gene signature related to cuproplasia was identified as a potential biomarker for predicting cancer patients’ survival. These findings provide novel insights into the effects of genes involved in cuproplasia on the molecular regulatory mechanisms of cancer development. Furthermore, the identified cuproplasia-related genes can be used by clinicians to understand which patients can benefit from using copper chelation therapy and identify novel combinatorial therapeutic strategies.

## Electronic supplementary material

Below is the link to the electronic supplementary material.

Additional file 1. Supplementary table 1 The 23 cancer types utilised in our analysis with corresponding number of samples obtained from TCGA (‘TCGA primary tumour’) and GTEx (‘GTEx normal tissue’). **Supplementary Table 2.** Copper related gene sets from the Molecular Signatures Database. **Supplementary Table 3.** 133-gene list of copper-metabolism related genes. **Supplementary Table 4.** Numbers of differentially expressed genes (DEGs) in cancer types. **Supplementary Table 5.** Sets of critical cuproplasia-related genes. **Supplementary Table 6.** Univariate Cox regression analysis for 13 CCGs identified in LGG.

Additional file 2. Supplementary Fig. 1 Survival curves for patient groups identified by using critical cuproplasia-related genes (CCGs). The survival analysis has been done by using the 18 up-regulated CCGs and the 12 down-regulated CCGs for each cancer type. The survival curves are presented in (**A**) ESCA, (**B**) GBM, (**C**) KICH, (**D**) KIRC, (**E**) KIRP, (**F**) LIHC, (**G**) LUSC, (**H**) OVCA, (**I**) PAAD, (**J**) PCPG, (**K**) PRAD, (**L**) STAD, (**M**) UCEC, and (**N**) UCS. **Supplementary Fig. 2. Expression profiles of CCG-related genes.** Heatmap of the (**A**) 18 up-regulated and (**B**) 12 down-regulated genes of patients as classified by Euclidean distances using gene expression values into 2 groups. **Supplementary Fig. 3. Systematic evaluation of cuproplasia-related pan-cancer gene signature using sigQC.** The radar plot in the centre shows the summary of the evaluation for the cuproplasia-related pan-cancer gene signature. The outer ring includes plots used to evaluate for standardisation, scoring metrics, compactness (i.e., intra-signature correlation), signature gene expression, and variability of the signature genes. These plots are located around the radar plot and are summarised by numeric values on the radar plot. The radar plot illustrates all the metrics of the signature in the pan-cancer dataset. **Supplementary Fig. 4. Mutation location of the top 10 mutated genes.** Lollipop diagram for the top 10 genes with the most pathogenic single nucleotide variants (SNVs). **(A)***ATP7A*, **(B)**, *CP*, **(C)***APP*, **(D)***TMPRSS6*, **(E)***DBH*, **(F)***ARF1*, **(G)***CYP1A1*, **(H)***ADAM10*, **(I)***AQP1*, and **(J)***GSK3B*. The x-axis is the amino acid location with the corresponding protein domains annotated. The y-axis is the number of mutations. The colours of the circles correspond to the type of mutation (Missense; green, Nonsense; red). The number beside the variant classification indicates the total number of mutations. **Supplementary Fig. 5. Survival analysis of mutant CCGs pan-cancer.** (**A**) *ADAM10*, (**B**) *AOC3*, (**C**) *AP1S1*, (**D**) *APP*, (**E**) *ARF1*, (**F**) *ATP7A*, (**G**) *CDK1*, (**H**) *CYP1A1*, (**I**) *DBH*, (**J**) *MAPT*, (**K**) *MT1X*, (**L**) *PRNP*, (**M**) *S100A12*, (**N**) *SLC11A2*, (**O**) *SNCA*, (**P**) *SORD*, (**Q**) *SP1*, (**R**) *TMPRSS6*, and (**S**) *XAF1*. **Supplementary Fig. 6. Cuproplasia-related LGG gene signature in the CGGA dataset** Survival curve (**A**) and gene expression heatmap (**B**) for the 3-gene signature most predictive of survival. Subtype 1 patients have high expression of *CDK1* and *CASP3* and low expression of *ALB*. Subtype 2 patients have low expression of *CDK1* and *CASP3* and high expression of *ALB*. Subtype 3 are all other patients. **Supplementary Fig. 7. Cuproplasia-related gene signatures as potential biomarkers for survival prediction.** Survival curves (top) and gene expression heatmaps (bottom) for cancer patients with (**A**) 8-gene signature when using the data of all pan-cancer patients, consisting of *CDK1, AP1S1, CASP3, MAP1LC3A, SNCA, TMPRSS6, MAPT*, and *GSK3B.* Subtype 1 are patients with low expression of *MAP1LC3A*, *SNCA*, and *MAPT* and high expression of *CDK1*, *AP1S1*, *CASP3*, *TMPRSS6*, and *GSK3B*. Subtype 2 are patients with high expression *MAP1LC3A*, *SNCA*, and *MAPT* and low expression of *CDK1*, *AP1S1*, *CASP3*, *TMPRSS6*, and *GSK3B.* Subtype 3 are all other patients. (**B**) 7-gene signature when using the data of pan-cancer patients and a half number of breast cancer patients only, consisting of *CDK1, AP1S1, CASP3, MAP1LC3A, SNCA, MAPT*, and *GSK3B.* Subtype 1 are patients with low expression of *MAP1LC3A*, *SNCA*, and *MAPT* and high expression of *CDK1*, *AP1S1*, *CASP3*, and *GSK3B*. Subtype 2 are patients with high expression *MAP1LC3A*, *SNCA*, and *MAPT* and low expression of *CDK1*, *AP1S1*, *CASP3*, and *GSK3B.* Subtype 3 are all other patients.

## Data Availability

The datasets analysed during the current study are available in the UCSC Xena repository (https://xenabrowser.net/datapages/) (Goldman et al. 2020) and CGGA database (http://www.cgga.org.cn/) (Liu et al. 2018b; Wang et al. 2015; Zhao et al. 2021).

## Code Availability

All code necessary to replicate these analyses is provided in the following GitHub repository https://github.com/CCI-BIO/Pan-cancerAnalysis. The version numbers of the R packages used in this study are listed in “Session information” section in Additional file 2.
