# Supplementary material for: A novel network-based method identifies a cuproplasia-related pan-cancer gene signature to predict patient outcome
Source: Hum Genet. 2024 Apr 20;143(9-10):1145–62. doi: 10.1007/s00439-024-02673-2 (PMC11485146; doi:10.1007/s00439-024-02673-2)
Supplement: Supplementary file 2 — Additional file 2. Supplementary Fig. 1 Survival curves for patient groups identified by using critical cuproplasia-related genes (CCGs). The survival analysis has been done by using the 18 up-regulated CCGs and the 12 down-regulated CCGs for each cancer type. The survival curves are presented in (A) ESCA, (B) GBM, (C) KICH, (D) KIRC, (E) KIRP, (F) LIHC, (G) LUSC, (H) OVCA, (I) PAAD, (J) PCPG, (K) PRAD, (L) STAD, (M) UCEC, and (N) UCS. Supplementary Fig. 2. Expression profiles of CCG-related genes. Heatmap of the (A) 18 up-regulated and (B) 12 down-regulated genes of patients as classified by Euclidean distances using gene expression values into 2 groups. Supplementary Fig. 3. Systematic evaluation of cuproplasia-related pan-cancer gene signature using sigQC. The radar plot in the centre shows the summary of the evaluation for the cuproplasia-related pan-cancer gene signature. The outer ring includes plots used to evaluate for standardisation, scoring metrics, compactness (i.e., intra-signature correlation), signature gene expression, and variability of the signature genes. These plots are located around the radar plot and are summarised by numeric values on the radar plot. The radar plot illustrates all the metrics of the signature in the pan-cancer dataset. Supplementary Fig. 4. Mutation location of the top 10 mutated genes. Lollipop diagram for the top 10 genes with the most pathogenic single nucleotide variants (SNVs). (A)ATP7A, (B), CP, (C)APP, (D)TMPRSS6, (E)DBH, (F)ARF1, (G)CYP1A1, (H)ADAM10, (I)AQP1, and (J)GSK3B. The x-axis is the amino acid location with the corresponding protein domains annotated. The y-axis is the number of mutations. The colours of the circles correspond to the type of mutation (Missense; green, Nonsense; red). The number beside the variant classification indicates the total number of mutations. Supplementary Fig. 5. Survival analysis of mutant CCGs pan-cancer. (A) ADAM10, (B) AOC3, (C) AP1S1, (D) APP, (E) ARF1, (F) ATP7A, (G) CDK1, (H) [file 439_2024_2673_MOESM2_ESM.pdf]

Human Genetics journal

A novel network-based method identifies a cuproplasia-related pan-cancer gene signature to predict patient outcome

Vu Viet Hoang Pham[1,2], Toni Rose Jue[1,2], Jessica Bell[1,2], Fabio Luciani[2], Filip Michniewicz[2], Giuseppe Cirillo[3], Linda Vahdat[4], Chelsea Mayoh[1,5,+], and Orazio Vittorio[1,2,+,*]

[1] Children's Cancer Institute, Lowy Cancer Research Centre, UNSW, Kensington, NSW, Australia

[2] School of Biomedical Sciences, UNSW Sydney, Kensington, NSW, Australia

[3] Department of Pharmacy, Health and Nutritional Sciences, University of Calabria, Rende, Italy

[4] Dartmouth–Hitchcock Medical Center: Lebanon, New Hampshire, US

[5] School of Clinical Medicine, UNSW Medicine & Health, UNSW Sydney, Kensington, NSW, Australia

[+] Joint senior authors

* To whom correspondence should be addressed. Tel: +61 2 9385 1557; Email: orazio.vittorio@unsw.edu.au

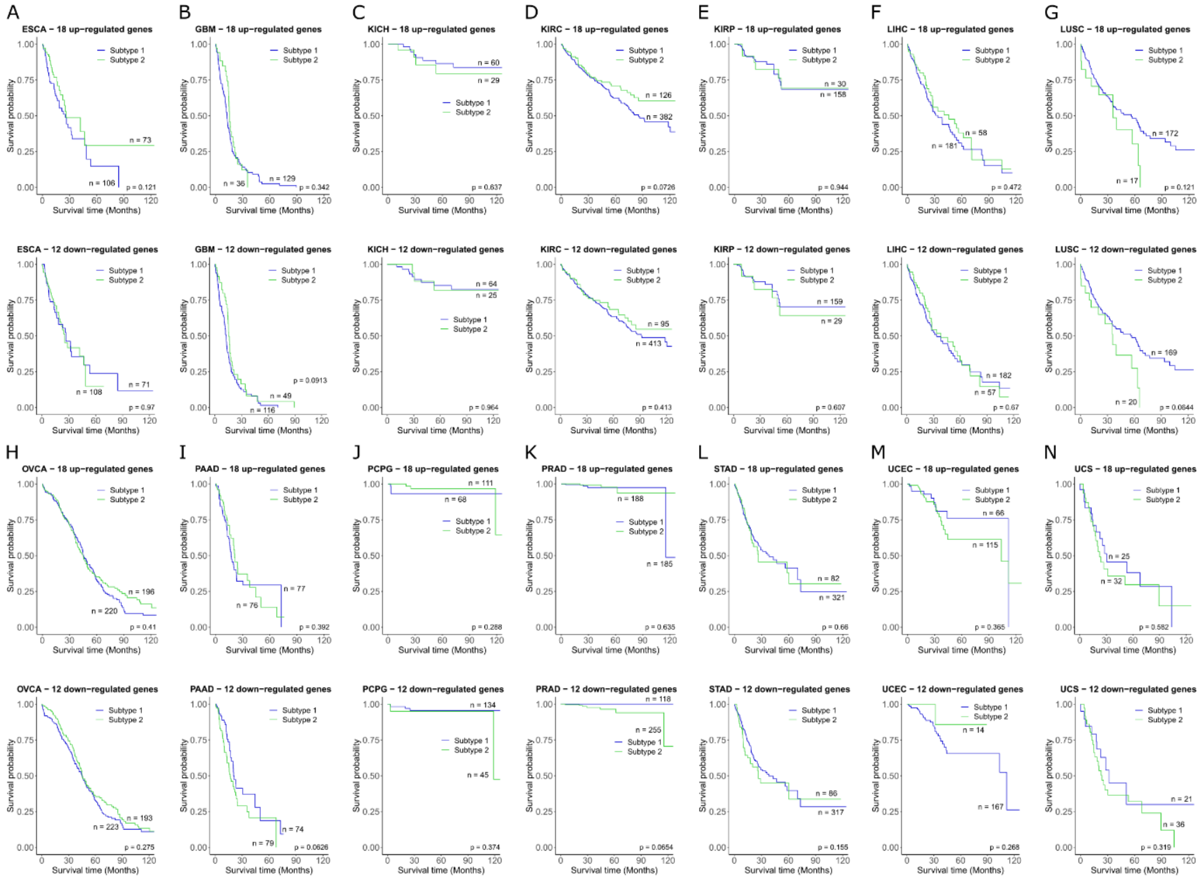

Supplementary Figure 1. Survival curves for patient groups identified by using critical cuproplasia-related genes (CCGs). The survival analysis has been done by using the 18 up-regulated CCGs and the 12 down-regulated CCGs for each cancer type**.** The survival curves are presented in (**A**) ESCA, (**B**) GBM, (**C**) KICH, (**D**) KIRC, (**E**) KIRP, (**F**) LIHC, (**G**) LUSC, (**H**) OVCA, (**I**) PAAD, (**J**) PCPG, (**K**) PRAD, (**L**) STAD, (**M**) UCEC, and (**N**) UCS.

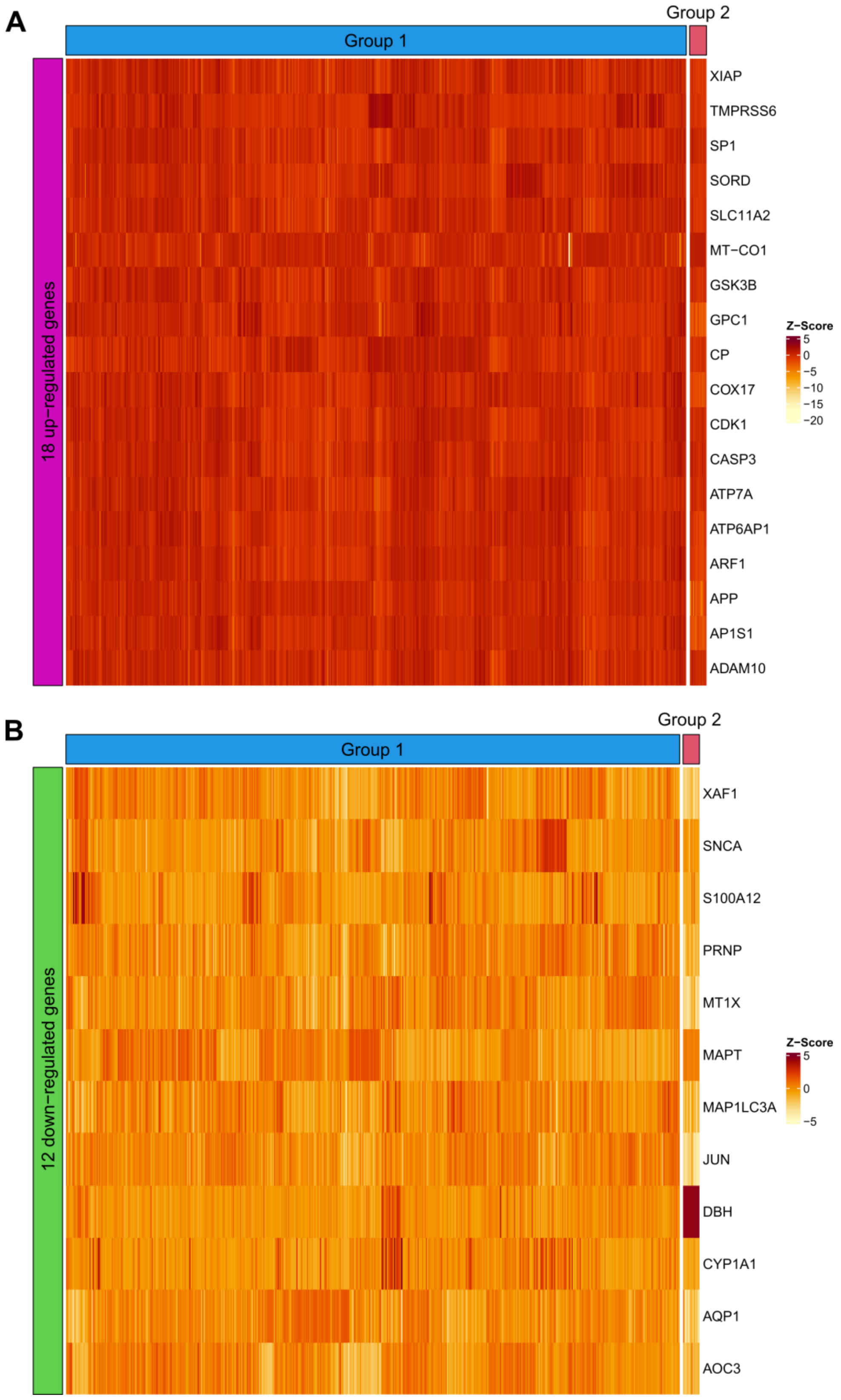

Supplementary Figure 2. Expression profiles of CCG-related genes. Heatmap of the (**A**) 18 up-regulated and (**B**) 12 down-regulated genes of patients as classified by Euclidean distances using gene expression values into 2 groups.

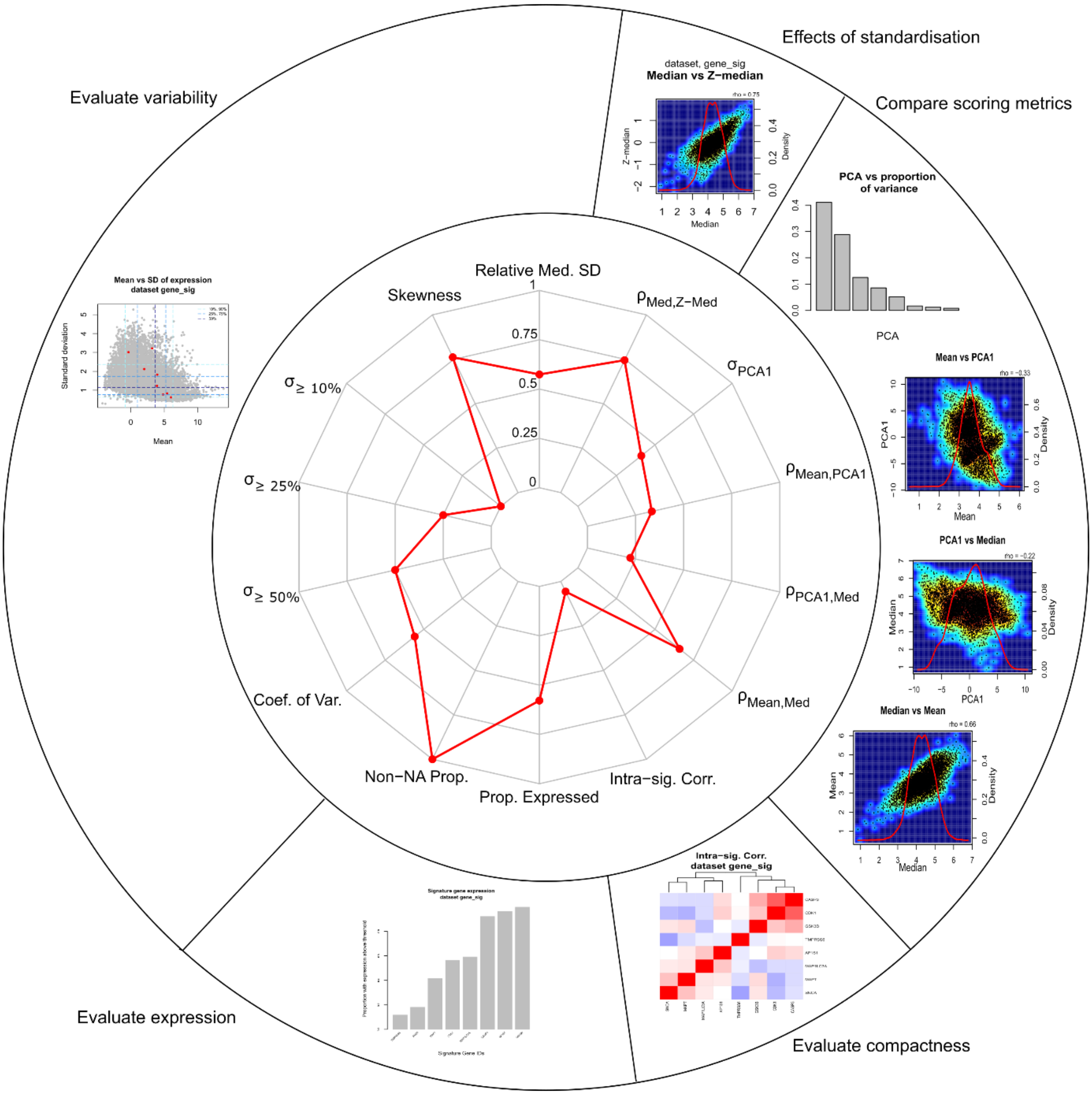

Supplementary Figure 3. Systematic evaluation of cuproplasia-related pan-cancer gene signature using sigQC. The radar plot in the centre shows the summary of the evaluation for the cuproplasia-related pan-cancer gene signature. The outer ring includes plots used to evaluate for standardisation, scoring metrics, compactness (i.e., intra-signature correlation), signature gene expression, and variability of the signature genes. These plots are located around the radar plot and are summarised by numeric values on the radar plot. The radar plot illustrates all the metrics of the signature in the pan-cancer dataset.

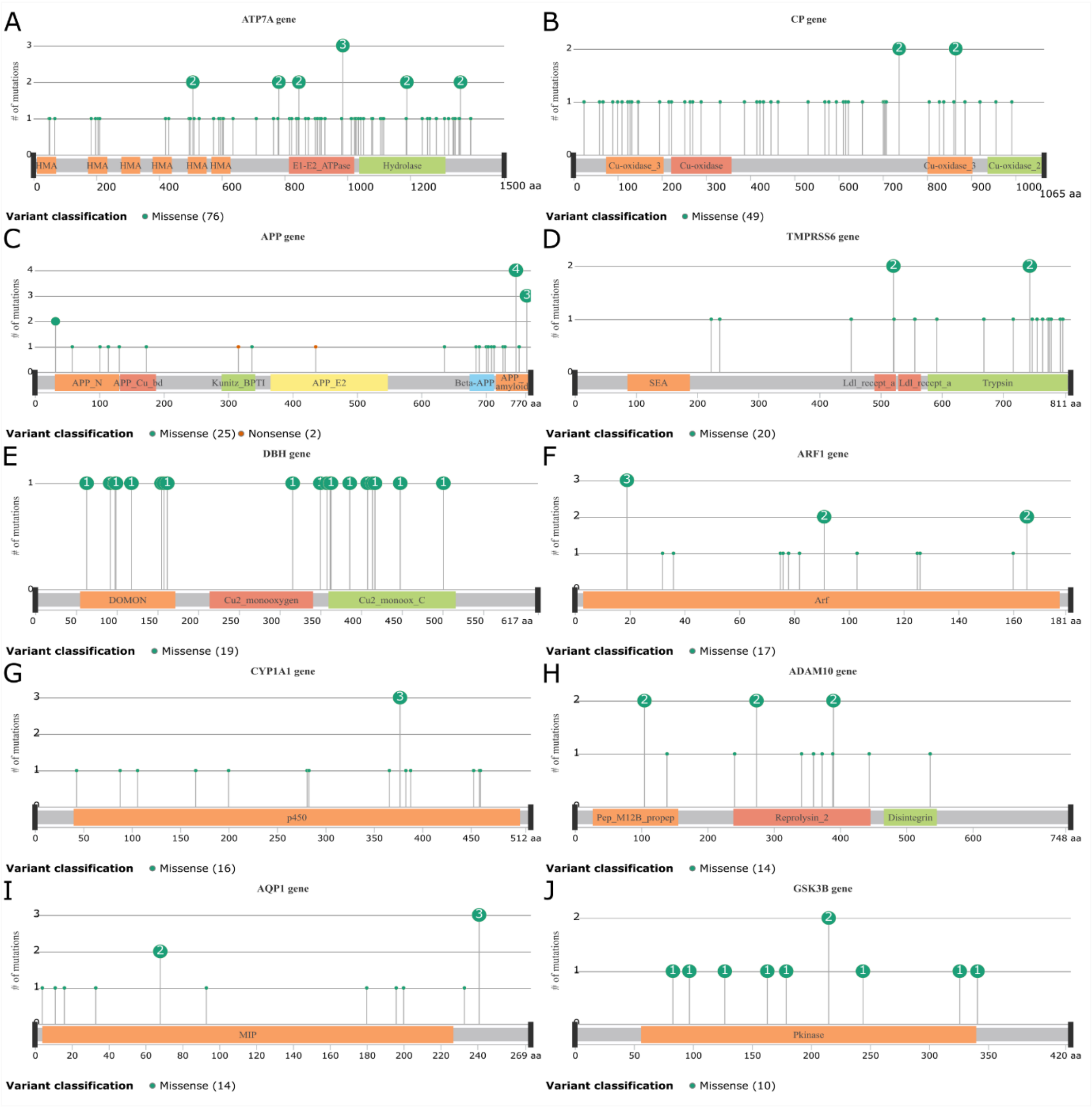

Supplementary Figure 4. Mutation location of the top 10 mutated genes. Lollipop diagram for the top 10 genes with the most pathogenic single nucleotide variants (SNVs). **(A)** *ATP7A*, **(B)**, *CP*, **(C)** *APP*, **(D)** *TMPRSS6*, **(E)** *DBH*, **(F)** *ARF1*, **(G)** *CYP1A1*, **(H)** *ADAM10*, **(I)** *AQP1*, and **(J)** *GSK3B*. The x-axis is the amino acid location with the corresponding protein domains annotated. The y-axis is the number of mutations. The colours of the circles correspond to the type of mutation (Missense; green, Nonsense; red). The number beside the variant classification indicates the total number of mutations.

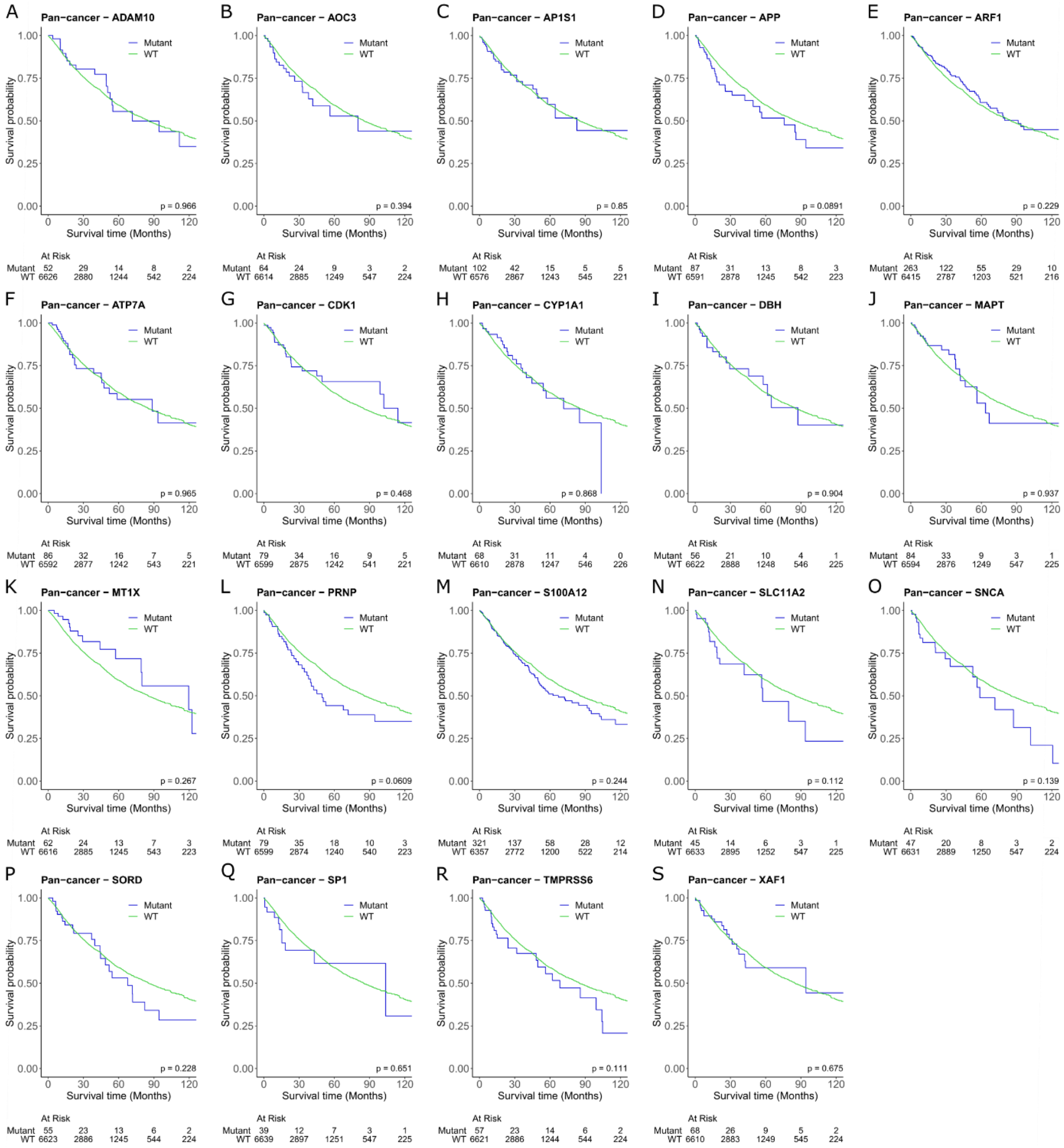

Supplementary Figure 5. Survival analysis of mutant CCGs pan-cancer. (**A**) *ADAM10*, (**B**) *AOC3*, (**C**) *AP1S1*, (**D**) *APP*, (**E**) *ARF1*, (**F**) *ATP7A*, (**G**) *CDK1*, (**H**) *CYP1A1*, (**I**) *DBH*, (**J**) *MAPT*, (**K**) *MT1X*, (**L**) *PRNP*, (**M**) *S100A12*, (**N**) *SLC11A2*, (**O**) *SNCA*, (**P**) *SORD*, (**Q**) *SP1*, (**R**) *TMPRSS6*, and (**S**) *XAF1*.

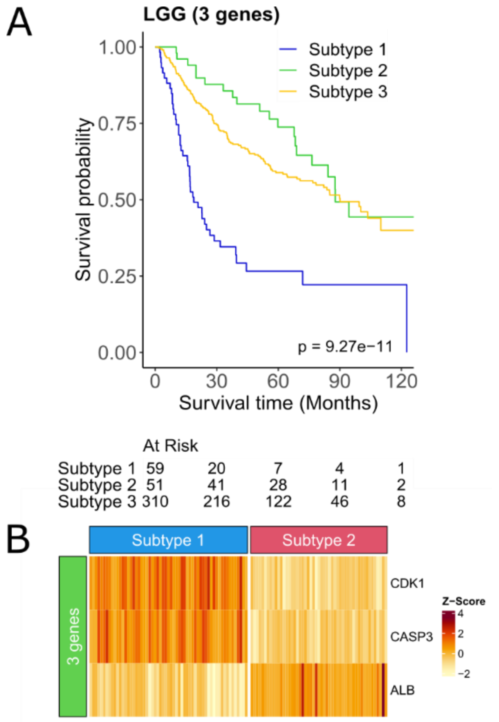

Supplementary Figure 6. Cuproplasia-related LGG gene signature in the CGGA dataset Survival curve (**A**) and gene expression heatmap (**B**) for the 3-gene signature most predictive of survival. Subtype 1 patients have high expression of *CDK1* and *CASP3* and low expression of *ALB*. Subtype 2 patients have low expression of *CDK1* and *CASP3* and high expression of *ALB*. Subtype 3 are all other patients.

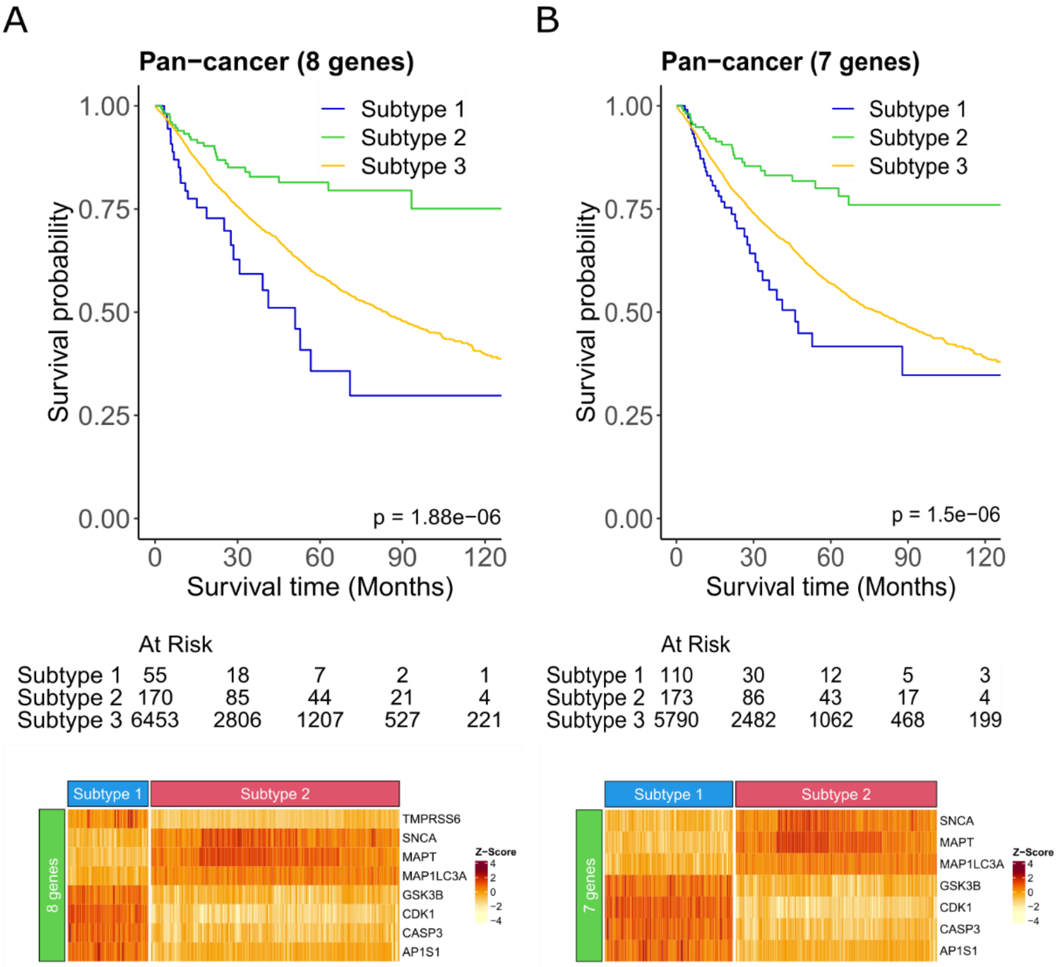

Supplementary Figure 7. Cuproplasia-related gene signatures as potential biomarkers for survival prediction. Survival curves (top) and gene expression heatmaps (bottom) for cancer patients with (**A**) 8-gene signature when using the data of all pan-cancer patients, consisting of *CDK1*, *AP1S1*, *CASP3*, *MAP1LC3A*, *SNCA*, *TMPRSS6*, *MAPT*, and *GSK3B*. Subtype 1 are patients with low expression of *MAP1LC3A*, *SNCA*, and *MAPT* and high expression of *CDK1*, *AP1S1*, *CASP3*, *TMPRSS6*, and *GSK3B*. Subtype 2 are patients with high expression *MAP1LC3A*, *SNCA*, and *MAPT* and low expression of *CDK1*, *AP1S1*, *CASP3*, *TMPRSS6*, and *GSK3B*. Subtype 3 are all other patients. (**B**) 7-gene signature when using the data of pan-cancer patients and a half number of breast cancer patients only, consisting of *CDK1*, *AP1S1*, *CASP3*, *MAP1LC3A*, *SNCA*, *MAPT*, and *GSK3B*. Subtype 1 are patients with low expression of *MAP1LC3A*, *SNCA*, and *MAPT* and high expression of *CDK1*, *AP1S1*, *CASP3*, and *GSK3B*. Subtype 2 are patients with high expression *MAP1LC3A*, *SNCA*, and *MAPT* and low expression of *CDK1*, *AP1S1*, *CASP3*, and *GSK3B*. Subtype 3 are all other patients.

Session information

R version 4.2.2 (2022-10-31)

Platform: x86_64-pc-linux-gnu (64-bit)

Running under: Rocky Linux 8.8 (Green Obsidian)

Matrix products: default

BLAS/LAPACK: /apps/z_install_tree/linux-rocky8-ivybridge/gcc-12.2.0/intel-oneapi-mkl-2022.2.1-mbdgclv5pa6o7p62mgo5v4xxwu2pa2k5/mkl/2022.2.1/lib/intel64/libmkl_gf_lp64.so.2

```
Random number generation:
 RNG:     Mersenne-Twister
 Normal:  Inversion
 Sample:  Rounding

locale:
 [1] LC_CTYPE=en_AU.UTF-8       LC_NUMERIC=C
 [3] LC_TIME=en_AU.UTF-8        LC_COLLATE=en_AU.UTF-8
 [5] LC_MONETARY=en_AU.UTF-8    LC_MESSAGES=en_AU.UTF-8
 [7] LC_PAPER=en_AU.UTF-8       LC_NAME=C
 [9] LC_ADDRESS=C               LC_TELEPHONE=C
[11] LC_MEASUREMENT=en_AU.UTF-8 LC_IDENTIFICATION=C

attached base packages:
[1] stats4    grid      stats     graphics  grDevices utils     datasets
[8] methods   base

other attached packages:
 [1] org.Hs.eg.db_3.16.0       sna_2.7-2
 [3] statnet.common_4.9.0      network_1.18.2
 [5] GGally_2.2.0              reticulate_1.28
 [7] RTCGAToolbox_2.28.4       glmnet_4.1-7
 [9] Matrix_1.5-3              plotly_4.10.1
[11] viridis_0.6.2             viridisLite_0.4.1
[13] gridExtra_2.3             ggpubr_0.6.0
[15] reshape_0.8.9             xtable_1.8-4
[17] EnsDb.Hsapiens.v79_2.99.0 ensembldb_2.22.0
[19] AnnotationFilter_1.22.0   GenomicFeatures_1.50.4
[21] AnnotationDbi_1.60.0      GenomicRanges_1.50.2
[23] GenomeInfoDb_1.34.9       IRanges_2.32.0
[25] S4Vectors_0.36.2          biomaRt_2.54.0
```

```
[27] patchwork_1.1.2           ggsurvfit_0.3.0
[29] circlize_0.4.15           ComplexHeatmap_2.15.4
[31] scales_1.2.1              plyr_1.8.8
[33] openxlsx_4.2.5.2          g3viz_1.1.5
[35] ezcox_1.0.2               SNFtool_2.3.1
[37] CancerSubtypes_1.24.0     NMF_0.26
[39] Biobase_2.58.0            BiocGenerics_0.44.0
[41] cluster_2.1.4             rngtools_1.5.2
[43] registry_0.5-1            sigclust_1.1.0.1
[45] maftools_2.16.0           reshape2_1.4.4
[47] lubridate_1.9.2           forcats_1.0.0
[49] stringr_1.5.0             dplyr_1.1.2
[51] purrr_1.0.1               readr_2.1.4
[53] tidyr_1.3.0               tibble_3.2.1
[55] tidyverse_2.0.0           ggplot2_3.4.4
[57] TCGAbiolinks_2.25.3       wesanderson_0.3.6
[59] survival_3.5-3            edgeR_3.40.2
[61] limma_3.54.2              RColorBrewer_1.1-3
[63] data.table_1.14.8

loaded via a namespace (and not attached):
  [1] utf8_1.2.3                 tidyselect_1.2.0
  [3] RSQLite_2.3.0              htmlwidgets_1.6.1
  [5] AnVIL_1.10.2               BiocParallel_1.32.5
  [7] munsell_0.5.0              codetools_0.2-19
  [9] DT_0.27                    miniUI_0.1.1.1
 [11] withr_2.5.0                colorspace_2.1-0
 [13] filelock_1.0.2             knitr_1.42
 [15] ggsignif_0.6.4             MatrixGenerics_1.10.0
 [17] GenomeInfoDbData_1.2.9     cBioPortalData_2.10.3
 [19] bit64_4.0.5                downloader_0.4
 [21] coda_0.19-4                vctrs_0.6.3
 [23] generics_0.1.3             lambda.r_1.2.4
 [25] xfun_0.37                  timechange_0.2.0
 [27] BiocFileCache_2.6.1        R6_2.5.1
 [29] doParallel_1.0.17          clue_0.3-64
 [31] RJSONIO_1.3-1.8            locfit_1.5-9.7
 [33] bitops_1.0-7               cachem_1.0.7
 [35] DelayedArray_0.24.0        promises_1.2.0.1
 [37] BiocIO_1.8.0               gtable_0.3.1
```

```
 [39] prettyGraphs_2.1.6          rlang_1.1.1
 [41] GlobalOptions_0.1.2         splines_4.2.2
 [43] rtracklayer_1.58.0          rstatix_0.7.2
 [45] lazyeval_0.2.2              impute_1.72.3
 [47] broom_1.0.4                 abind_1.4-5
 [49] BiocManager_1.30.20         yaml_2.3.7
 [51] RaggedExperiment_1.22.0     backports_1.4.1
 [53] httpuv_1.6.9                tools_4.2.2
 [55] TCGAutils_1.18.0            gridBase_0.4-7
 [57] ellipsis_0.3.2              DNAcopy_1.72.3
 [59] MultiAssayExperiment_1.24.0 Rcpp_1.0.10
 [61] progress_1.2.2              zlibbioc_1.44.0
 [63] RCurl_1.98-1.10             prettyunits_1.1.1
 [65] GetoptLong_1.0.5            SummarizedExperiment_1.28.0
 [67] magrittr_2.0.3              futile.options_1.0.1
 [69] ProtGenerics_1.30.0         matrixStats_0.63.0
 [71] hms_1.1.2                   TCGAbiolinksGUI.data_1.18.0
 [73] mime_0.12                   XML_3.99-0.13
 [75] shape_1.4.6                 compiler_4.2.2
 [77] crayon_1.5.2                htmltools_0.5.4
 [79] later_1.3.0                 tzdb_0.3.0
 [81] DBI_1.1.3                   formatR_1.14
 [83] dbplyr_2.3.1                rappdirs_0.3.3
 [85] car_3.1-2                   cli_3.6.0
 [87] parallel_4.2.2              pkgconfig_2.0.3
 [89] GenomicAlignments_1.34.0    RCircos_1.2.2
 [91] ExPosition_2.8.23           xml2_1.3.3
 [93] foreach_1.5.2               ggstats_0.5.1
 [95] XVector_0.38.0              rvest_1.0.3
 [97] digest_0.6.31               ConsensusClusterPlus_1.62.0
 [99] Biostrings_2.66.0           forestmodel_0.6.2
[101] restfulr_0.0.15             curl_5.0.0
[103] shiny_1.7.4                 Rsamtools_2.14.0
[105] rjson_0.2.21                lifecycle_1.0.3
[107] GenomicDataCommons_1.22.1   jsonlite_1.8.4
[109] carData_3.0-5               futile.logger_1.4.3
[111] rapiclient_0.1.3            alluvial_0.1-2
[113] fansi_1.0.4                 pillar_1.9.0
[115] lattice_0.20-45             KEGGREST_1.38.0
[117] fastmap_1.1.1               httr_1.4.5
```

```
[119] glue_1.6.2          zip_2.2.2
[121] png_0.1-8           iterators_1.0.14
[123] bit_4.0.5           stringi_1.7.12
[125] blob_1.2.3          memoise_2.0.1
```
